# Supplementary material for: Factors influencing adoption of oral health promotion by antenatal care providers in Moyo district, North-Western Uganda
Source: PLoS One. 2023 Apr 25;18(4):e0272238. doi: 10.1371/journal.pone.0272238 (PMC10128944; doi:10.1371/journal.pone.0272238)
Supplement: Attachment — Submitted filename: PONE-D-22-18056_R1_reviewed.pdf [file pone.0272238.s004.pdf]

PLOS ONE

Factors influencing adoption of oral health promotion by antenatal care providers in Moyo district, North-Western Uganda

--Manuscript Draft--

**Manuscript Number:**	PONE-D-22-18056R1
**Article Type:**	Research Article
**Full Title:**	Factors influencing adoption of oral health promotion by antenatal care providers in Moyo district, North-Western Uganda
**Short Title:**	Factors influencing adoption of oral health promotion by antenatal care providers in Moyo district.
**Corresponding Author:**	Patrick Madrama Lulu, MPH<br>Clarke International University<br>Kampala, UGANDA
**Keywords:**	
**Abstract:**	Background : Oral health promotion during pregnancy is an important global public health and basic human right issue related to quality of life. Several statements and guidelines have been published emphasizing the need for improved oral health care of pregnant mothers, prenatal care providers have missed this critical opportunity. In this study, we assessed factors influencing adoption of oral health promotion by Antenatal care providers.<br>Materials and Methods: A descriptive cross-sectional study design that employed both quantitative and qualitative data collection methods and analysis. 152 samples determined using Yamane's 1967 and stratified sampling technique was used. Three FGDs and six KI interviews were held. Univariate, bivariate and multivariate analyses were done using SPSS (20.0) and ATLAS Ti for qualitative analysis.<br>Results: Adoption of OHP was low 28% (42). Factors influencing adoption of oral health promotion were age of respondents(p=0.032*), level of care of health facility (p=0.004*), good understanding between dentists and ANC providers (aOR=0.283, 95%CI=0.084-0.958, p= 0.042*), availability of practice guideline for OHP in ANC (aOR=0.323, 95%CI=0.108-0.958, p= 0.043*), number of years at work (p=0.084), being knowledgeable (aOR=2.143, 95%CI=0.864-5.311, p= 0.100), having skills to advance OHP(aOR=0.734, 95%CI=0.272-1.984, p= 0.542), Management being good at influencing new practices (aOR=00.477.734, 95%CI=0.227-2.000, p= 0.477). More emphasis on national and local of oral health issues, continuous staff training on oral health, dissemination of National oral health policy (NOHP) were some of key issues that emerged from the qualitative results.<br>Conclusion: Adoption of OHP was low. This was attributed to age, the number of years spent at work, level of health facility, having good understanding between dentists and ANC providers, availability of practice guidelines on prenatal oral care, dissemination of National oral health policy, continuous staff training. We recommend the current NOHP to be reviewed, develop prenatal OHC guidelines, enhance the capacity of ANC providers through training, collaboration with dentists and launch official adoption of OHP.
**Order of Authors:**	Patrick Madrama Lulu, MPH
	Miisa Nanyingi, MBchB,MSHSM
**Response to Reviewers:**	Patrick Madrama Lulu,<br>Clarke International University,<br>P.0. Box 7782, Kampala- Uganda<br>14th .11.2022<br><br>Dear Emily Chenette,<br>Editor in Chief, Plos One Journal<br><br>Ref: Response to Reviewers for “Factors influencing adoption of oral health promotion by antenatal care providers in Moyo district, North-Western Uganda.”

Powered by Editorial Manager® and ProduXion Manager® from Aries Systems Corporation

Thank you for taking time to review our manuscript that was submitted to you, thank you for the comments. Below are the responses to the reviewers.

Section Reviewers' CommentResponses
Abstractyou may need to insert the OR and CIF for the first 2 factors as wellOR and CIF have been inserted for the first 2 factors.
Delete aa, have been deleted from aOR
Background
This sentence better to come after the second sentence in the introduction as it is continuation regarding oral health followed by the sentence starting with Antenatal care
The suggested sentence has been inserted after the second sentence in the introduction as suggested by the reviewers.
Study Population and settings
Exclusion criteria is to be revised!
as a general principle exclusion is to exclude the already included (fulfilling the inclusion criteria) but with some characteristics they are to be excluded.
Exclusion criteria has been revised following general principles.

Study Variables
What was the logic behind applying these cut-off points?
I would suggest to use the mean score as the cut-off point!The researchers have now clearly indicated the values for mean scores for each cut off point.
what was the cut-off point in these two variablesThe cut-off point for these two variables have now been clearly indicated.
Study Design
Could you please elaborate on the equation by adding the numbers used in the equation e.g. population size
The formula for calculating the sample size has been elaborated as suggested by the reviewers and population size indicated.
Data Analysis
what was the level of significance used?
This has been adjusted.
Results
This is not shown the table 1Has been adjusted as required.
what is the expected level and the actual level measured?It was an error, expected levels have been removed.
This table would be better presented in two columns the second containing both frequency and parentage!This table 1 has been adjusted as suggested by reviewers.
This should be 69.7
the total adds to 100.2%This value has been adjusted in table 1, as required.
This should be 54%This has as well been adjusted as required.
You need to mention the OR and CIOR and CI have been added.
ONLY this age-group was significant others were not
check the OR and CI AND p-values as well!This has been adjusted as required.
this comparison is wrong!!!
the reference group is 31-40 years' experience both groups are compared to the last group of experience (they are not significant look at te CI) REVISEThis has been revised.
This comparison is wrong the reference group is HC IVThis has been adjusted.
Wrong interpretation of the UOR!!
REVISEThe interpretation has been revised.
compared to those had skills!!This comparison has been adjusted.
Mention the livelihood between the two groups UOR three times, eight times and twiceThe likelihood between the two groups have been revised.
This sentence belong to the discussion than the results sectionThe sentence has been removed from results section.
These results depicted in figure 3 NOT oneThis has been adjusted as required.
These results are depicted in figure 1.Has been adjusted as required.
What was the CIThis has been revised in table 3.
Strength and Limitations

Data analyses does not control for any bias!

Powered by Editorial Manager® and ProduXion Manager® from Aries Systems Corporation

	This has been revised.<br>References<br>Any abbreviation for this journal!<br>Abbreviation for the journals have been indicated as required.<br><br><br>Yours Sincerely<br><br>Patrick Madrama Lulu<br>Phone: +256 782 98 04 51<br>Email:Lulu.madrama@gmail.com<br>Consultant Research Ethics Committee/Lecturer, Clarke International University
**Additional Information:**	
**Question**	**Response**
**Financial Disclosure**<br><br>Enter a financial disclosure statement that describes the sources of funding for the work included in this submission. Review the submission guidelines for detailed requirements. View published research articles from *PLOS ONE* for specific examples.<br><br>This statement is required for submission and **will appear in the published article** if the submission is accepted. Please make sure it is accurate.<br><br>**Unfunded studies**<br>Enter: *The author(s) received no specific funding for this work.*<br><br>**Funded studies**<br>Enter a statement with the following details:<br>• Initials of the authors who received each award<br>• Grant numbers awarded to each author<br>• The full name of each funder<br>• URL of each funder website<br>• Did the sponsors or funders play any role in the study design, data collection and analysis, decision to publish, or preparation of the manuscript?<br>• **NO** - Include this sentence at the end of your statement: *The funders had no role in study design, data collection and analysis, decision to publish, or preparation of the manuscript.*<br>• **YES** - Specify the role(s) played.<br><br><br><br>* typeset	The authors received no specific funding for this work.
**Competing Interests**	The authors have declared that no competing interests exist.

Powered by Editorial Manager® and ProduXion Manager® from Aries Systems Corporation

Use the instructions below to enter a competing interest statement for this submission. On behalf of all authors, disclose any competing interests that could be perceived to bias this work—acknowledging all financial support and any other relevant financial or non-financial competing interests.<br><br>This statement is required for submission and **will appear in the published article** if the submission is accepted. Please make sure it is accurate and that any funding sources listed in your Funding Information later in the submission form are also declared in your Financial Disclosure statement.<br><br>View published research articles from *PLOS ONE* for specific examples.<br><br>**NO authors have competing interests**<br><br>Enter: *The authors have declared that no competing interests exist.*<br><br>**Authors with competing interests**<br><br>Enter competing interest details beginning with this statement:<br><br>*I have read the journal's policy and the authors of this manuscript have the following competing interests: [insert competing interests here]*<br><br>* typeset	
**Ethics Statement**<br><br>Enter an ethics statement for this submission. This statement is required if the study involved:<br><br>• Human participants<br>• Human specimens or tissue<br>• Vertebrate animals or cephalopods<br>• Vertebrate embryos or tissues	This study was approved by Clarke International University Ethical Committee.

Powered by Editorial Manager® and ProduXion Manager® from Aries Systems Corporation

• Field research<br><br>Write "N/A" if the submission does not require an ethics statement.<br><br>General guidance is provided below. Consult the submission guidelines for detailed instructions. **Make sure that all information entered here is included in the Methods section of the manuscript.**	

Powered by Editorial Manager® and ProduXion Manager® from Aries Systems Corporation

**Format for specific study types**<br><br>**Human Subject Research (involving human participants and/or tissue)**<br>• Give the name of the institutional review board or ethics committee that approved the study<br>• Include the approval number and/or a statement indicating approval of this research<br>• Indicate the form of consent obtained (written/oral) or the reason that consent was not obtained (e.g. the data were analyzed anonymously)<br><br>**Animal Research (involving vertebrate animals, embryos or tissues)**<br>• Provide the name of the Institutional Animal Care and Use Committee (IACUC) or other relevant ethics board that reviewed the study protocol, and indicate whether they approved this research or granted a formal waiver of ethical approval<br>• Include an approval number if one was obtained<br>• If the study involved *non-human primates*, add *additional details* about animal welfare and steps taken to ameliorate suffering<br>• If anesthesia, euthanasia, or any kind of animal sacrifice is part of the study, include briefly which substances and/or methods were applied<br><br>**Field Research**<br><br>Include the following details if this study involves the collection of plant, animal, or other materials from a natural setting:<br>• Field permit number<br>• Name of the institution or relevant body that granted permission	
**Data Availability**<br><br>Authors are required to make all data underlying the findings described fully available, without restriction, and from the time of publication. PLOS allows rare exceptions to address legal and ethical concerns. See the PLOS Data Policy and FAQ for detailed information.	Yes - all data are fully available without restriction

A Data Availability Statement describing where the data can be found is required at submission. Your answers to this question constitute the Data Availability Statement and **will be published in the article**, if accepted.<br><br>**Important:** Stating 'data available on request from the author' is not sufficient. If your data are only available upon request, select 'No' for the first question and explain your exceptional situation in the text box.<br><br>Do the authors confirm that all data underlying the findings described in their manuscript are fully available without restriction?	
**Describe where the data may be found in full sentences. If you are copying our sample text, replace any instances of XXX with the appropriate details.**<br><br>• If the data are **held or will be held in a public repository**, include URLs, accession numbers or DOIs. If this information will only be available after acceptance, indicate this by ticking the box below. For example: *All XXX files are available from the XXX database (accession number(s) XXX, XXX.).*<br>• If the data are all contained **within the manuscript and/or Supporting Information files**, enter the following: *All relevant data are within the manuscript and its Supporting Information files.*<br>• If neither of these applies but you are able to provide **details of access elsewhere**, with or without limitations, please do so. For example:<br><br>*Data cannot be shared publicly because of [XXX]. Data are available from the XXX Institutional Data Access / Ethics Committee (contact via XXX) for researchers who meet the criteria for access to confidential data.*<br><br>*The data underlying the results presented in the study are available from (include the name of the third party*	N/A

and contact information or URL).<br>• This text is appropriate if the data are owned by a third party and authors do not have permission to share the data.<br><br>* typeset	
Additional data availability information:	

Manuscript Click here to access/download;Manuscript;Manuscript.docx

Factors influencing adoption of oral health promotion by antenatal care providers in Moyo district, North-Western Uganda

Patrick Madrama Lulu (MPH)[1], Miisa Nanyingi (MBChB, MSHSM)[2]

[1] Faculty of Health Sciences, Uganda Martyrs University, P.o. Box 5498, Kampala –Uganda.
Current address: Clarke International University, P.o. Box 7782, Kampala-Uganda.
[2]Faculty of Health Sciences, Uganda Martyrs University, P.o. Box 5498, Kampala -Uganda.

Corresponding Author: Patrick Madrama Lulu; Email:lulu.madrama@gmail.com; https://orcid.org/0000-0002-2838-8532

Key Words: *Adoption, Oral Health, Oral Health Promotion, Oral Hygiene, Antenatal care providers, Healthy Public Policy, perinatal, Periodontal Disease, UMU*

Abstract

Background: Oral health promotion(OHP) during pregnancy is an important global public health and basic human right issue related to quality of life. Several statements and guidelines have been published emphasizing the need for improved oral health care of pregnant mothers, prenatal care providers have missed this critical opportunity. In this study, we assessed factors influencing adoption of oral health promotion by Antenatal care providers.

Materials and Methods: A descriptive cross-sectional study design that employed both quantitative and qualitative data collection methods and analysis. 152 samples determined using Yamane's 1967 and stratified sampling technique was used. Three FGDs and six KI interviews were held. Univariate, bivariate and multivariate analyses were done using SPSS (20.0) and ATLAS Ti for qualitative analysis.

Results: Adoption of OHP was low 28% (42). Factors influencing adoption were age of respondents (OR=0.066, 95%CI=0.009-0.465, p= 0.006*), level of care of health facility (OR=0.050, 95%CI=0.008-0.322, p= 0.002*), good understanding between dentists and ANC providers (OR=0.283, 95%CI=0.084-0.958, p= 0.042*), availability of practice guideline for OHP in ANC (OR=0.323, 95%CI=0.108-0.958, p= 0.043*), number of years at work (p=0.084), being knowledgeable (OR=2.143, 95%CI=0.864-5.311, p= 0.100), having skills to advance OHP(OR=0.734, 95%CI=0.272-1.984, p= 0.542), Management being good at influencing new practices (OR=00.477.734, 95%CI=0.227-2.000, p= 0.477). More emphasis on national and local of oral health issues, continuous staff training on oral health, dissemination of National oral health policy (NOHP) were some of key issues that emerged from the qualitative results.

Conclusion: Adoption of OHP was low. This was attributed to age, number of years spent at work, level of health facility, having good understanding between dentists and ANC providers, availability of practice guidelines, dissemination of National oral health policy, continuous staff training. We recommend the current NOHP to be reviewed, develop prenatal OHC guidelines, enhance the capacity of ANC providers through training, collaboration with dentists and launch official adoption of OHP.

Background

Oral disease is one of the most important public health issues in the world with significant socio-economic impacts [1, 2, 3, 4]. Among pregnant women, the common oral health diseases are periodontal diseases with a high prevalence rate [5]. Oral health is a basic human right fundamental to people's quality of life [6], achieving oral health goals would require health systems being supported by enabling actions in education, improved nutrition and hygiene [7]. Antenatal care is the care provided by skilled healthcare professionals to pregnant women and adolescent girls to ensure the best health conditions for both mother and baby during pregnancy and its components include; risk identification, prevention and management of pregnancy-related or concurrent diseases including health education and health promotion [8]. Pregnancy has an impact on oral related quality of life [9]. Pregnancy provides an opportunity for women to initiate new healthy behaviours, including oral health practices, dental and prenatal care providers have missed this critical opportunity to promote oral health [10]. Some of the missed opportunities for oral health promotion include; oral health education, oral health examination and referral for dental services when mothers are receiving antenatal care.

Globally, several statements and guidelines have been published emphasizing the need for improved oral health care of pregnant mothers, because, hormonal changes during pregnancy combined with neglected oral hygiene tend to increase the incidence of oral diseases including gingivitis [11]. The United States of America, Department of Health and Human Services highlighted the need for partnerships between dentists and other health professionals, including nurses and midwives and these recommendations have resulted in the development of evidence-based practice guidelines for oral health care during pregnancy and early childhood for all health professionals including prenatal care providers (American Academy of Pediatric Dentistry, 2009; California Dental Association Foundation, American College of Obstetricians and Gynecologists, 2010; National Maternal and Child Oral Health Resource Center, 2008; [12].

In low and middle-income countries, the prevalence of oral diseases continues to increase, moreover, tooth decay is rapidly increasing among adults and there will be a huge burden of this health problem in the future if sustainable programs are not put in place [13, 14]. The good oral health of a pregnant woman should be considered to be of utmost importance for dental practitioners and antenatal care providers [15, 16, 17, 18]. Integration of Oral health into primary care has been implemented in some health care systems to reduce the burden of oral health disease and to improve access to oral health care, especially for disadvantaged populations and communities [19, 20, 21]. Asia developed a strategy in which emphasis was put on the integration of oral health promotion and other disease prevention strategies [22], various countries have implemented preventive strategies to maintain the oral health of mothers during pregnancy [23].

Adopting an integrated approach to healthcare would achieve better outcomes for patients with oral diseases and several policy recommendations have been made including the adoption of oral health in all policies, strengthening inter-professional collaboration, and inclusion of oral health in the curriculum for all healthcare professionals [24, 25]. In addition, the WHO global policy for the improvement of oral health showed that the promotion of oral health is a cost-effective strategy to reducing the burden of oral diseases and maintaining oral health and quality of life. Oral Health services should focus on prevention and early diagnosis [26, 27].

In Sub-Saharan Africa, there is an increased prevalence of dental diseases, resulting from increased consumption of sugars and inadequate exposure to fluorides [28], with an increased burden of periodontal diseases among pregnant women in Mali [29, 30, 31], while pregnant mothers in Tanzania had increased burden of bleeding gum, dental pain, tooth decay and swollen gums [32]. Nigeria's National oral health policy indicated the need for oral health to be included as a component of health promotion and must be provided in antenatal care services [33, 34] and emphasis has been placed on integrating oral health promotion in maternal and child care among African Countries [35, 36].

In Uganda, the prevalence of oral diseases among women was the highest at 42.4% compared to the general population [37], 67.0% of pregnant mothers having periodontal diseases [38], 86.0% postpartum mothers with plague deposits [39]. Uganda has emphasised that appropriate promotion of oral health requires integration of oral health policy elements, strategies and policies of all sectors to impact community health, including maternal and child health [40]. In the Moyo district, there is limited data on the level and factors associated with the adoption of Oral health promotion by antenatal care providers.

Materials and Methods

Study Population and settings

This cross sectional study on adoption of oral health promotion was done among antenatal care providers working in general hospital, Health Centre IV and health centre IIIs within Moyo district between august 2017 to October 2017.

The inclusion criteria required that, for the ANC providers to participate in the study, they should be health workers working at ANC units, those who had ever worked at the ANC unit, including all Nurses, Midwives, Clinical Officers and medical doctors within the selected health facilities who consented to participate in the study. We excluded all Nurses, Midwives, Clinical Officers, Gynaecologists, paediatricians, general medical doctors and those who had never worked at the ANC department before the study and all ANC providers who had spent less than six months at work at the time this study was done.

Study variables

Adoption of Oral Health Promotion as a dependent variable was measured using 24 items of practices related to oral health care services by antenatal care providers, with a total score of mean of 18 (75%, SD 3.2) and a greater proportion of ANC providers had not adopted oral health promotion during ANC.

Knowledge and attitude influencing adoption of oral health promotion: 11 items were used to measure the level of knowledge, with a mean score of correct answers 7 (64%, SD 2.2) For measuring attitude of the respondents, 6 items were used, with a mean score 4 (66%, SD 0.98).

Capacity of Health facilities to provide oral health promotion activities: 16 items were used to measure the capacity through binary scale (no or yes) and 11 items used to measure the **strategies required to influencing adoption of oral health promotion**, through binary scale (no or yes) there were inadequate strategies to influencing oral health promotion by ANC providers.

Study Design

A descriptive cross-sectional study was used where both quantitative and qualitative methods of data collection and analysis were employed. A concurrent data collection approach was used including information and the interpretation of overall results [41], this design was used because of the shorter data collection period and the need to provide a comprehensive analysis of the research problem.

152 samples were determined using Yamane's (1967) formula $\mathbf{n = \frac{N}{1+N(e)^2}}$, Where n = sample size, N = Number of ANC providers that include Nurses, Midwives, Clinical Officers and Medical Doctors in the selected health facilities within Moyo district (245), e = Level of precision or sampling error (0.05).and a stratified sampling technique was used. Three FGDs and six KI interviews were held. Univariate, bivariate and multivariate analyses were done using SPSS (20.0) for quantitative analysis and ATLAS Ti for qualitative analysis.

Data Analysis

We analysed Quantitative data using Microsoft Excel 2010 and SPSS 20. Chi-square statistics were computed to check for statistically significant differences in the parameters between the dependent and independent variables (Univariate, bivariate and multivariate analysis were performed in SPSS version 20.0). Logistic regression was done to obtain strength of association between categorical dependent and independent variables and statistically significant items at this level were then analysed using multivariate logistic regression to obtain adjusted conclusions for the finding of the study. Qualitative data were transcribed verbatim, coded and organised in themes in ATLAS.ti. Triangulation was done to incorporate both the quantitative and qualitative data obtained from respondents.

Ethical Considerations

This study was conducted in accordance with the Helsinki declaration guideline, a waiver was granted by International Health Sciences University Research Ethics Committee (IHSU-REC) (IHSU-REC/004). Prior to the waiver, administrative clearance was done by Uganda Martyrs University Scientific Review Committee, a written consent was obtained from Moyo District Health Office (DHO), verbal consent from the medical superintendent of Moyo hospital, in-charges of the Dental unit, Antenatal care department and various health facilities under study and finally from participants under study.

Results

Demographic Characteristics of the respondents

Our study considered a total of 152 ANC providers, mean age 36, range 37 years, a standard deviation of 10.49, of which, 57(37.5%) were 37 years and above, 92(60.5%) females, 106(69.9%) had worked between 1-10 years, 82(53.9%) working HC IIIs, 67(44.1%) Nurses and 82(53.9%) were Certificate holders. (See **Table *1***).

Table 1: Demographic Characteristics of respondents

Variables	Number (%)<br>N=152(%)	
**Age**		
22-26<br>27-31<br>32-36<br>37 & above	29(19.1)<br>33(21.7)<br>33(21.7)<br>57(37.5)	19.1<br>21.7<br>21.7<br>37.5
**Gender**		
Male<br>Female	60(39.5)<br>92(60.5)	39.5<br>60.5
**Number of years at work**		
1-10 years	106(69.7)	69.7
11-20 years	23(15.1)	15.1
21-30 years	8(5.3)	5.3
31-40 years	15(9.9)	9.9
**Level of Health Facility**		
Hospital	40(26.3)	26.3
HCIV	30(19.7)	19.7
HCIII	82(54)	54
**Professional Cadre**		
Nurses	67(44.1)	44.1
Midwives	35(23.0)	23.0

Double trained Nurse/midwives	24(15.8)	15.8
Clinical Officers	2013.2)	13.2
Medical doctors	6(3.9)	3.9
**Professional Qualification**		
Certificate	82(53.9)	53.9
Diploma	60 (39.5)	39.5
Bachelor's Degree	10(6.6)	6.6

how was the adoptation measured??

Level of adoption of Oral Health Promotion by ANC Providers

42 (28%) of the respondents out of 152 had adopted oral health promotion for pregnant mothers. Although individual practices of ANC providers appeared to be low, the management and officer in charge of the MCH programme noted that adoption or integration of oral health promotion in ANC would be vital for overcoming some of the challenges of poor oral health, its outcome on pregnancy and the child (See **Figure *1***).

> *"There is need to adopt or integrate oral health promotion into ANC services to overcome some of the challenges created by the poor oral health of the pregnant mothers" (KI 1).*

More emphasis supporting the need for adoption of Oral Health promotion by ANC providers showed that adoption is likely to reduce heart diseases among pregnant mothers;

> *"Of late there are increased cases of heart diseases…… originating from the dental issues as bacteria tend to move to the bloodstreams and later on move to the heart valves without knowing, adopting oral health promotion will have a possibility of reducing such challenges in the future" KI 2).*

> *"It will be cheaper adopting oral health promotion into ANC services compared to when it handled as a separate program" (FGD 1).*

Figure 1:Level of adoption of oral Health promotion by ANC providers

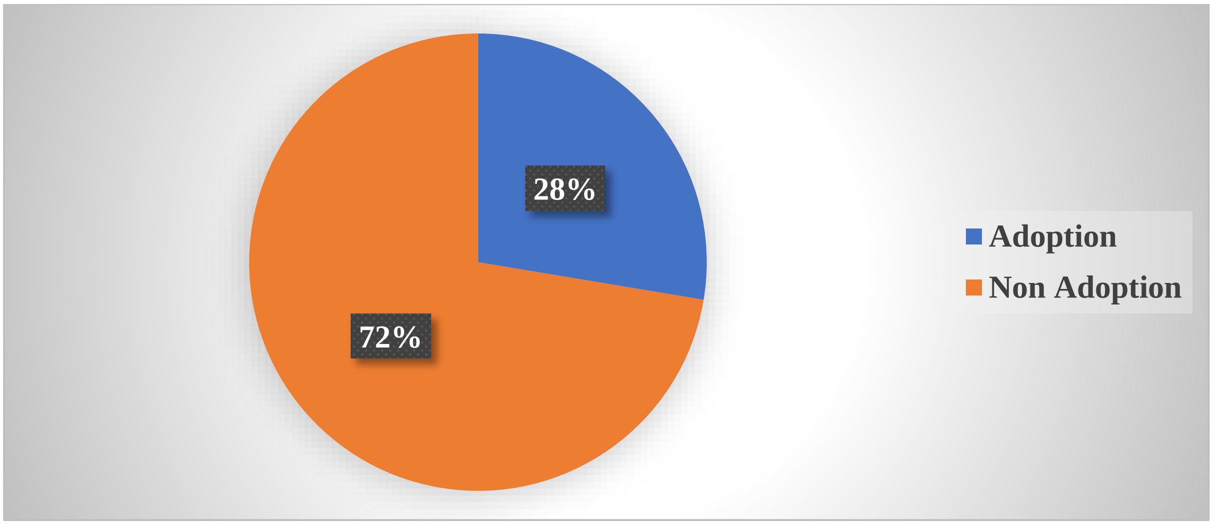

Factors influencing adoption of Oral Health Promotion by ANC Providers

In our multivariate analysis, age of respondents in years 22-26 (aOR=0.066, 95%CI=0.009-0.465, p= 0.006*), level of care of health facility (aOR=0.050, 95%CI=0.008-0.322, p= 0.002*), good understanding between dentists and ANC providers (aOR=0.283, 95%CI=0.084-0.958, p= 0.042*), availability of practice guideline for OHP in ANC (aOR=0.323, 95%CI=0.108-0.958, p= 0.043*), worked 1-10 years (p=0.011*), were statistically significant and associated with adoption of oral health promotion by ANC providers. However, general number of years at work (aOR= 25.241, 95CI =2.075-307.041, p=0.084), being knowledgeable (aOR=2.143, 95%CI=0.864-5.311, p= 0.100), having skills to advance OHP in ANC (aOR=0.734, 95%CI=0.272-1.984, p= 0.542), Management good at influencing new practices (aOR=00.477.734, 95%CI=0.227-2.000, p= 0.477) were not statistically significantly associated with adoption oral health promotion by ANC providers (see **Table *2***).

> *"Top management at the national level is not good at influencing new policies and guidelines, there is no dissemination of the National Oral health policy, if dental health issues are not marketed by the authorities, no one will pay attention and it will continue to be neglected, and yet adoption will lead to a reduction on missed opportunities, maximizing resources – one staff offering all that is needed and improving service delivery" (KI 1).*

In our univariate analysis, female ANC providers were more likely to adopt oral health promotion compared to their male counterparts. Respondents aged between 22-26 years were less likely to adopt oral health promotion during prenatal care compared to those aged 37 years and above. Respondents who had been working for the last 21-30 years were 14 times more likely to adopt oral health promotion compared to response who had for 31-40 years (see **Table *2***).

In terms of the level of health facilities, respondents working at the health centre IV were less likely to adopt oral health promotion compared to those working at HC IIIs.

Knowledgeable respondents were four times more likely to adopt oral health promotion compared to those that were not knowledgeable and respondents who had no skills to advance OHP in ANC were less likely to adopt oral health promotion compared to respondents who had no skills in advancing OHP for prenatal care.

Respondents who said that management was good at influencing new practices, were more likely to adopt oral health promotion compared to those who said that management was not good at influencing new practices. Having no good understanding between dentists and ANC providers less likely contributed to the adoption of OHP, and having no practice guidelines for OHP by ANC providers less likely led to the adoption of oral health promotion (See **Table *2***).

> *"Even if the staff members make recommendations for some dental items, the oral health department issues are always pushed aside and not taken care of" (KI 3).*
>
> *The whole district only offers extraction within government health facilities, other services are in a private dental clinic (KI 4).*
>
> *We do not have oral health policies for ANC and there is no local guideline that is put in place yet. We do not have the National Oral Health policy even in our dental department and I have never seen it before" (KI 2).*
>
> *There is a need for training of ANC providers on oral health promotion for pregnant mothers (FGD 1).*

The ANC providers were also asked to raise issues that should be included in the oral health promotion guideline. More than half 95 (62.5%) of the respondents mentioned almost all responses and these were; prevention of oral diseases and this was followed by oral health education 93 (61.2%), Oral health education 93 (61.6%), ways of improving access to oral healthcare 91(60.3%) and assessment of pregnant women's oral health 88 (58.3%) (See **Figure *2***).

Figure 2:Multiple responses showing issues to be included in oral health promotion guideline for ANC

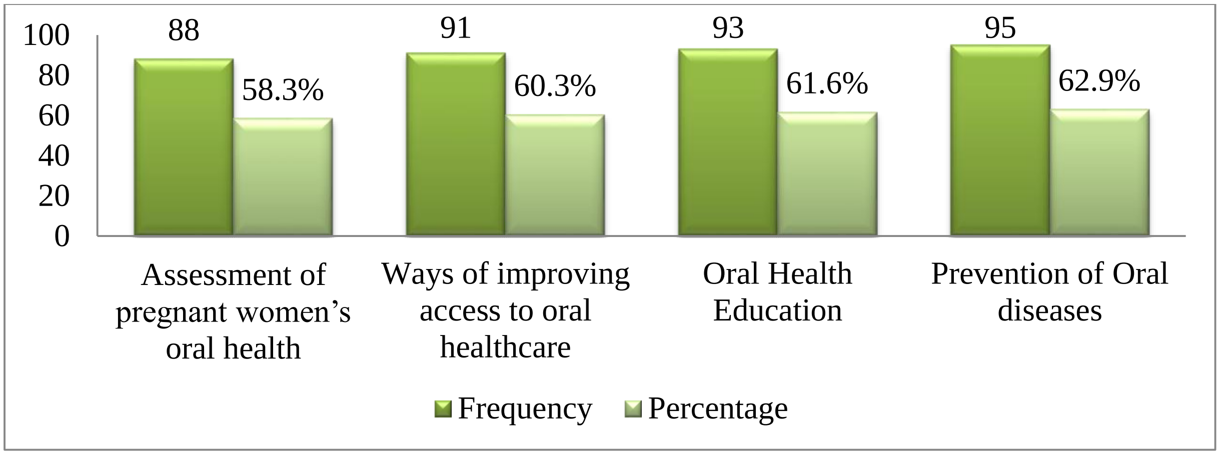

In line with participants' knowledge on the kind of preventive oral care pregnant women should receive the majority 124 (89.9%) of the respondents mentioned how to maintain oral hygiene, 57(41.3%) of the ANC providers mentioned education on nutrition, less than half 45(32.6%) and 29(21.0%) of the ANC providers mentioned oral health assessment as well as referral, only 2(1.4%) mentioned other preventive care services as the kind of preventive services pregnant women should receive (See **Figure *3***).

Figure 3:Knowledge on the kind of preventive oral care pregnant women should receive

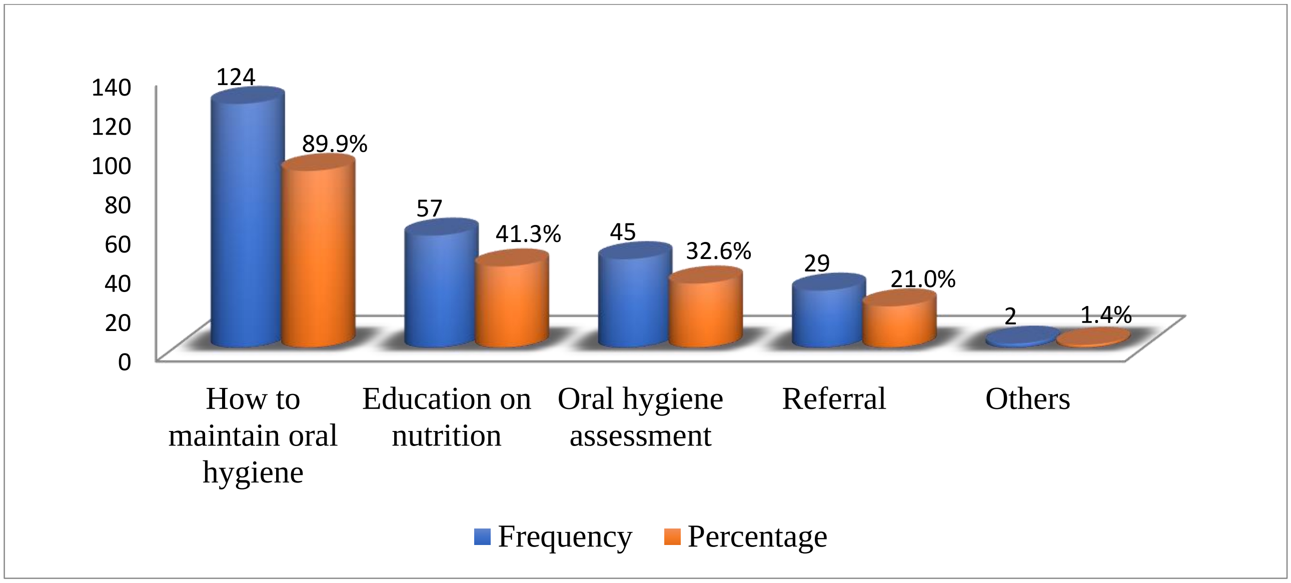

In line with the knowledge of respondents on the safety of obtaining dental radiographs during pregnancy, the majority 125 (82.2%) of the respondents were not aware that it is safe to obtain dental radiographs in pregnant women while only 26 (17.1%) of the respondents said obtaining dental radiograph in pregnant women is safe (See **Figure *4***).

Figure 4:Awareness on the safety of obtaining dental radiographs during pregnancy

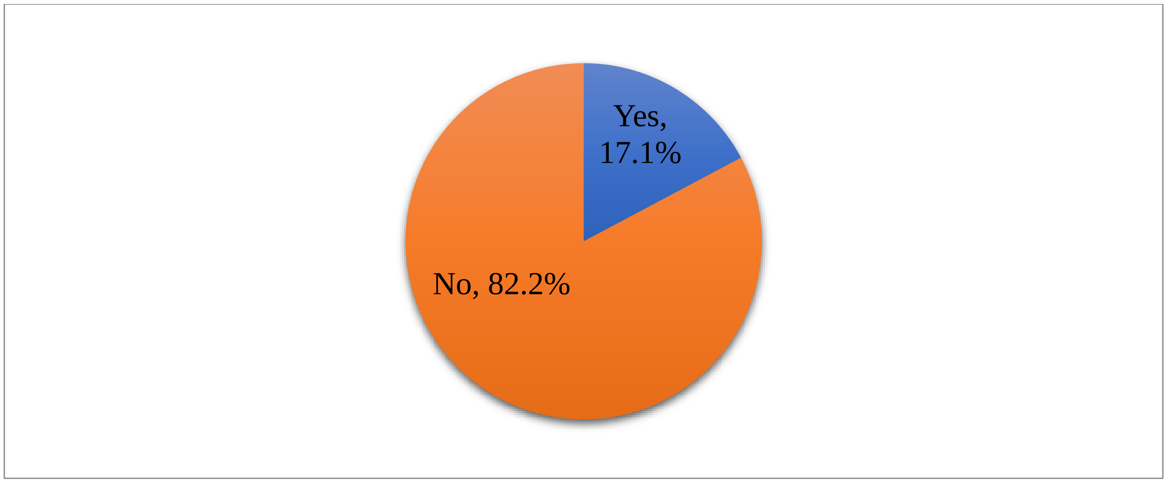

Table 2:Factors influencing adoption of oral health promotion by ANC providers in Moyo district, north-western Uganda

	Adoption of Oral Health Promotion by ANC Providers		Univariate Logistic Regression Analysis		Multivariate Logistic Regression Analysis	
**Variables**	**Adoption (%)**	**Non-Adoption (%)**	**UOR (95%CI)**	**P-value**	**AOR (95%CI)**	**P-value**
**Age in years**						
22-26	3	26	0.184(0.050-0.679)	0.011*	0.066(0.009-0.465)	**0.006***
27-31	8	25	0.509(0.195-1.327)	0.167	0.148(0.029-0.741)	**0.020***
32-36	2	24	0.597(0.235-1.517)	0.278	0.130(0.026-0.642)	0.084
37 & above	22	35	1		1	
**Number of years at work**						
1-10 years	1	14	5.273(0.663-41.920)	0.116	25.241(2.075-307.041)	**0.011***
11-20 years	29	17	7.467(0.825-67.573)	0.074	8.205(0.749-89.852)	0.085
21-30 years	8	15	14.000(1.200-163.367)	0.035*	12.064(0.775-187.885)	0.075
31-40 years	4	4	1		1	
**Level of Care**						
Hospital	2	28	0.827(0.366-1.869)	0.647	0.317(0.097-1.034)	0.057*
HC IV	12	28	0.138(0.031-0.621)	0.010*	0.050(0.008-0.322)	**0.002***
HCIII	28	54	1		1	
**Knowledge**						
Knowledgeable	7	49	4.016(1.642-9.823)	0.002*	2.143(0.864-5.311)	0.1000
Not Knowledgeable	35	61	1		1	
**Have skills to advance OHP in ANC**						
No	12	54	0.415(0.193-0.893)	0.024*	0.734(0.272-1.984)	0.542
Yes	30	56	1		1	
**Management is good at influencing new practices**						
No	7	44	0.300(0.122-0.735)	0.008*	0.477(0.227-2.000	0.477
Yes	35	66	1		1	
**A good understanding exists between dentists and ANC providers**						
No	6	47	0.223(0.087-0.674)	0.002*	0.283(0.084-0.958)	**0.042***
Yes	36	63	1		1	
**Practice guidelines for OHP in ANC are available**						
No	28	54	0.482(0.229-1.013)	0.054*	0.323(0.108-0.958)	**0.043***
Yes	14	56	1		1	

Discussion

Adoption of Oral Health Promotion by Respondents

In this study, adoption of oral health promotion by ANC providers was low 42 (28%) of 152. Similarly, a study conducted in Australia among antenatal care providers showed that very few ANC providers were involved in practices that promote oral health among pregnant women [42]. Adoption of oral health by ANC providers would play a significant role not only in the lives of pregnant mothers and also in their babies [43]. Maternal oral health behaviour could be shaped during antenatal care [44], promotion of oral health among pregnant women by Nurses would be feasible and need to be incorporated into the first antenatal booking visit and also recognized that oral health promotion was within their scope of practice [45].

Respondents aged between 22-26 years, 27- 31 years, and 32-36 years were less likely to adopt oral health promotion during prenatal care compared to those aged 37 years and above. There was a statistically significant relationship between age and adoption of oral health promotion. This may be due to experiences gained over the years about oral health. Contrary to the findings of our study, another study noted that age is significantly and negatively associated with workability [46], job satisfaction among Nurses is also related to age [47].

Moreover, the number of years one spent at work improves occupational competence, consists of expertise, skills, and knowledge acquired through experience, training, and education, people's work and are influenced by previous work experiences [48]. Our study noted that respondents who had been working for the last 21-30 years were 14 times more likely to adopt oral health promotion compared to response who had for 31-40 years, who were 6 times more likely compared to those for 27- 31 years, and 8 times more likely to those who had for 22-26 years respectively. Similarly, [49] noted in their study that, there was an association between years spent at work and adoption of oral health promotion, Nurses 'experiences are closely related to the occurrence of adverse events [50]. However, Positive work

environments are important in achieving patient safety, quality care and favourable patient outcomes [51].

Similarly, experience give Nurses' exposure to different patient or health-related conditions, including different clinical and preventive scenarios that would eventually contribute to increased knowledge, critical thinking and technical skills [52]. In general, job performance among nurses is motivated by years of experience [53]. However, it has also been noted that age and length of time in service within ANC do not influence knowledge of oral healthcare for prenatal mothers [54].

In terms of the level of the health facility where respondents were working, our study found that those working at the district general hospital were less likely to adopt oral health promotion compared to respondents working at HC IIIs. However, working at a general hospital contributed to 82.7% adoption compared to working at HCIV which only contributed to 13.8% adoption of oral health promotion by ANC providers. This may be because most of the care of HCIIIs in the district are offered by generally Nurses, Midwives and Clinical officers, ANC at this level of facility handles all aspects of preventive services including oral health education and promotion. The general hospital and HCIVs may be overwhelmed by several conditions because of referrals from lover facilities.

In our study, less than half 56(36.8%) of the respondents were knowledgeable about OHP in ANC and those knowledgeable were four times more likely to adopt oral health promotion compared to those that were not knowledgeable. Similarly, Nurses and Midwives had insufficient knowledge of oral healthcare [54]. Respondents in our study were not knowledgeable about the fact that several physiological changes take place among pregnant women and these have an effect on oral health, pregnancy and brings about several changes in the oral cavity [55]. Our finding was similar to the study in Australia which noted that ANC providers had limited knowledge on prenatal oral healthcare [49].

In terms of knowledge on the safety of dental procedures during pregnancy, our study found that ANC providers were not knowledgeable about the safety of different dental services that pregnant women

should receive, including the use and safety of local anaesthetics during pregnancy, teeth extraction, scaling and Root planning and Root Canal. This knowledge about the safety of dental procedures during pregnancy should be addressed to improve service utilization and oral health promotion among pregnant mothers. Similarly, a study by [55] found a knowledge gap among gynecologists about the safety of some of the dental procedures for pregnant mothers. Local anesthetics lidocaine commonly used during dental care are safe, although, with some negligible adverse effect [56], some of these effects may include immune response-mediated allergic reactions and others that are unrelated to the immune response and these allergic responses are very rare in practice [57].

About the safety of obtaining dental radiographs in pregnant women, the majority 125 (82.2%) of the ANC providers were not aware that it is safe to obtain dental radiographs in pregnant women and use of dental radiographs, if necessary, after the first trimester does not pose any risk to the developing fetus [58], however, it is recommended to use protective lead aprons and thyroid collar to shield the sensitive areas. This kind of misconception could however be overcome among ANC providers by continuous services professional training and collaboration among different professionals within the different health facilities.

In our study, respondents who had no skills to advance OHP in ANC were less likely to adopt oral health promotion compared to those who said they had skills in advancing OHP for prenatal care. This calls for the need to incorporate prenatal oral care in health higher institutions of learning and continuous professional education for ANC providers in practice. Health institutions have limited training for prenatal oral healthcare [59], adequate skills attained by health workers would enable them to perform desired tasks, more regular training and paying attention to discuss career development prospects would be of great value to advance their skills [60].

In addition, our study found that having a good understanding between dentists and ANC providers contributed to adoption. A study conducted in Australia found no interprofessional collaboration and

understanding between non-dentists and dentists [61]. An inter-professional collaboration education model for dental and medical providers are needed and the provision of an appropriate referral system for comprehensive clinical care of pregnant patients and accredited standards that encourage development and implementation [63, 64]. There is a need for collaboration between ANC providers with dentists to encourage all pregnant women to comply with the oral health professionals' recommendations regarding appropriate dental brushing techniques and the importance of dental visits [63]. However, Collaboration across organizational boundaries remains challenging due to power dynamics and trust that affect the strategic choices made by each health professional about whether to collaborate, with whom and to what level [64]. These decisions directly affect inter-professional relationships trust and respect can be fostered through a mix of interventions aimed at building personal relationships and establishing agreed rules that govern collaborative care and that are perceived as fair to all professions. Some of the factors that facilitate the integration of oral health are interdisciplinary education and collaborative practices between dental and other healthcare professionals [65]. Adoption of oral promotion requires coordination and cooperation between ANC providers and dentists [66]. Understanding and interprofessional collaboration improve professional output, through the provision of clarity around the meaning and definition of professional roles [67].

In terms of management, respondents who said management was not good at influencing new practices among ANC providers were less likely to adopt oral health promotion compared to management being good at influencing new practices. Poor leadership were a key factor leading to providers' weak workplace trust and contributed to often-poor quality services, driving a perverse cycle of negative patient-provider relations [71]. For leaders to have lasting influence, staff members should be involved in health policy development and some of the key participatory roles in health policy development include mentoring, supporting and developing future policymakers [72].

In addition, the Ottawa charter emphasized that all sectors at all levels be involved in formulating policy and direct them to be aware of the health consequences of their decisions and to accept their responsibilities for health, health promotion policy requires the identification of obstacles to the adoption of healthy public policies and ways of removing them, thus health policies must focus on making it easy for beneficiaries to make healthier choices [73]. Involvement of staff members would help them on what is in the policy and also improve on policy dissemination and implementation at all levels, avoiding situations wherein a district, some oral health professionals have not even seen the national oral health policy until it was presented to them during the research work.

Moreover, our study found that there is a need to develop oral health promotion guidelines for pregnant mothers and according to [49], many countries have developed oral health guidelines for pregnant mothers to help them improve the oral health of both pregnant mothers and the babies and the main barriers for ANC providers in promoting oral health was lack of practice guidelines on oral health care during pregnancy. There is a need for local and national health policy agenda in Uganda to incorporate the prevention of oral diseases during pregnancy [38]. Burke-Litwin model noted that, when staff members feel that organizational policies and procedures are favourable to them, they are motived to perform the required actions, implying that appropriate formulation and implementation of policies motivate staff members to perform required actions.

Study Strength and Limitations

Our study had several strengths firstly, the large sample size covering three quarters of the health workers and all categories of cadres of health workers in the district, secondly, our study was the first in Uganda with a focus on the adoption of oral health promotion by antenatal care providers, we involved research assistants who were well trained to create rapport with respondents, thirdly, we trained research assistants on data collection procedures, maintaining confidentiality, ensure correctness and consistency of recorded data. Fourth, all our research tools were fully pretested and checked for accuracy and

correctness, only filled questionnaires were included for analysis. However, our study could not be used to analyse behaviour or practices of the ANC providers over some time as it was a snapshot, limitation of confounding bias which was controlled by restricting participation to only those who had worked for at least six months and above and participation to only ANC provider who had ever worked or are working at ANC department in order to arrive at meaningful and conclusive results.

4.3 Conclusion

Adoption of OHP was low. Factors influencing the adoption of oral health promotion were, Age of respondents, the number of years at work, good understanding between dentists and ANC providers, and management being good at influencing new practices contributed to an increased level in the adoption of oral health promotion. However, knowledge gap, poor dissemination of National oral health policy and lack of prenatal oral health guidelines were some of the barriers to an increased level of adoption by antenatal care providers. We, therefore, recommend that the current NOHP should be reviewed, develop prenatal OHC guidelines, enhance the capacity of ANC providers through training, collaboration with dentists and launch official adoption of OHP.

Abbreviations

ANC: Antenatal care; aOR: Adjusted Odds Ratio; CI: Confidence Interval; MoH: Ministry of Health; FDG: Focus Group Discussion; KI: Key Informants; uOR: Unadjusted Odds Ratio; UMU: Uganda Martyrs University; HSSIP: Health Sector Strategic and Investment Plan; WHO: World Health Organization.

Acknowledgement

We are grateful to Uganda Martyrs University for awarding the primary author with a master degree in Public Health – Health Promotion. We thank International Health Sciences University (Currently, Clarke International University) research ethics committee for according us a waiver for our protocol. we appreciate all our research assistants for the support rendered during the process of data collection.

Authors Contribution

PML and MN conceptualised and designed the study. PML obtained the needed data, PML analysed the data and interpreted the results, PLM drafted and made the final manuscript, PLM and MN performed critical revisions of the manuscript. Both PML and MN approved the final manuscript.

Funding

There was no funding by any organization, this research was carried out under the supervision of Uganda Martyrs University Faculty of Health Sciences as a requirement for the award of masters of Public Health- Health Promotion for the first author.

Availability of data and materials

All relevant data are within the paper and its Supporting Information files.

Consent for publication

No application

Competing interests

The authors declare that they do not have any competing interests.

References

[1] Marcenes.W, Kassebaum.NJ, Bernabe.E, Flaxman.A, Neghavi.M, Lopez.A, Murray.CJL., "Global Burden of oral conditions in 1990-2010: A systematic Analysis.," *.J Dent Res.DOI:10.1177/0022034513490168,* vol. 92, no. 7, pp. 592-595, 2013.

[2] Kassebaum.N, Hernandez.C, Bailey.J, Hay.SI, James.S,Mokdad.A, Nixon.M., "Global, Regional, and National Levels and Trends in Burden of Oral Conditions from 1990 to 2017: A Systematic Analysis for the Global Burden of Disease 2017 Study," *J Dent Res,* 2020.

[3] WHO, "Oral health. Key facts," World Health Organization, Geneva, 2020.

[4] Kassebaum, N.J., Smith, A.G., Bernabé, E., Fleming, T.D., Reynolds, A.E., Vos, T., Murray, C.J.L., Marcenes, W. and GBD 2015 Oral Health Collaborators., "Global, regional, and national prevalence, incidence, and disability-adjusted life-years for oral conditions for 195 countries, 1990–2015: a systematic analysis for the global burden of diseases, injuries, and risk factors," *Journal of dental research,* vol. 96, no. 4, pp. 380-387, 2017.

[5] Salih.Y, Nasr.AM, Ahmed.ABA, Sharif.ME, Adam.I., “Prevalence of and risk factors for periodontal disease among pregnant women in an antenatal care clinic in Khartoum, Sudan,” *BMC Research Notes,* vol. 13, no. 147, pp. 1-5, 2020.

[6] Marchi.KS, Rinki.C, Shah.M, Dove.M, Terpak .C, Curtis.MP, Braveman.P., “Medical Provider Promotion of Oral Health and Women's Receipt of Dental Care During Pregnancy,” *Jmch.doi: 10.1007/s10995-018-02714-z. ,* vol. 23, no. 7, pp. 890-902, 2019.

[7] Martínez.GR & Albuquerque.A, “The right to oral health in the Liverpool Declaration,” *Rev. bioét. (Impr.),* vol. 25, no. 2, pp. 224-33, 2017.

[8] Muhoozi.GK, Atukunda.P, Skaare.AB, Willumsen.T, Diep.LM, Westerberg.AC, Iversen.PO., “Effects of nutrition and hygiene education on oral health and growth among toddlers in rural Uganda: follow-up of a cluster-randomised controlled trial,” *Trop Med Int Health,* vol. 23, no. 4, pp. 391-404, 2018.

[9] WHO, "WHO recommendations on antenatal care for a positive pregnancy experience.," World Health Organization, 2016.

[10] Fakheran.O, Keyvanara.M, Saied-Moallemi .Z, Khademi.A., “The impact of pregnancy on women’s oral health-related quality of life: a qualitative investigation,” *BMC Oral Health,* vol. 20, no. 294, pp. 1-11, 2020.

[11] Harnagea.H,Lamothe.L,Couturier.H,Esfandiari.S,Voyer.R,Charbouneau.A,Emami.R., “From theoretical concepts to policies and applied prgrams:the landscaepe of integration of oral health in primary care,” *BMC Oral Health,* vol. 18, no. 23, pp. 1-12, 2018.

[12] Northridge. ME, Kumar.A, Kaur.R., “Disparities in Access to Oral Health Care,” *Annual Review of Public Health,* p. 513–35, 3 January 2020.

[13] WHO, "Prevention is better than treatment," Bull World Health Organ, 2015.

[14] WHO, “Oral health. Key facts,” World Health Organization, Geneva, 2020.

[15] Ramamurthy.J & Irfana.F, "Assessment of knowledge and awareness about periodontal oral health among pregnant women-A Questionnaire study," *Int J Cur Res Rev,* vol. 9, no. 1, pp. 9-12, 2017.

[16] Yenen.Z & Ataçağ.T,, “Oral care in pregnancy,” *J Turk Ger Gynecol Assoc 2019,* vol. 20, no. 4, pp. 264-8, 2019.

[17] Fakheran.O, Keyvanara.M, Saied-Moallemi.Z, Khademi.A., “The impact of pregnancy on women’s oral health-related quality of life: a qualitative investigation,” *BMC Oral Health.https://doi.org/10.1186/s12903-020-01290-5,* vol. 20, no. 294, pp. 1-11, 2020.

[18] García-Pola.M, González-Díaz.A, García-Martín.JM., “Effect of a Preventive Oral Health Program Starting during Pregnancy: A Case-Control Study Comparing Immigrant and NativeWomen and Their Children,” *J.Environ. Res. Public Health,* Vols. 18,, no. 4096, pp. 1-14, 2021.

[19] Petersen.PE, Baez.RJ, Ogawa.H., "Global application of oral disease prevention and health promotion as measured 10 years after the 2007 World Health Assembly statement on oral health," *Community Dental and Oral Health Epidemiology,* vol. 48, no. 4, pp. 338-348, 2020.

[20] Emami.E, Harnagea.H, Girard.F, Charbonneau.A, Voyer.R, Bedos.CP, Chartier.M, Wootton.J, Couturier.Y., "Integration of oral health into primary care: a scoping review protocol," *BMJ Open doi:10.1136/bmjopen-2016-013807,* vol. 10, no. 1136, pp. 1-5, 2016.

[21] Braimoh.M, Ogunbodede.E, Adeniyi.A., "Integration of oral health into primary health care system: views of primary health care workers in Lagos State, Nigeria," *Journal of Public Health in Africa,* vol. 5, no. 328, pp. 35-39, 2014.

[22] WHO, "Formulating Oral Health strategy for South-East Asia. Report of a Regional Consultation," World Health Organization, South Asia; India, Chiang Mai, Thailand, 2009.

[23] Ajesh.G, Sousa.MS, Kong.AC, Blinkhorn.A, Norrie.TP, Foster.J, Dahlen. HG, Ajwani.S, Johnson.M., "Effectiveness of preventive dental programs offered to mothers by non-dental professionals to control early childhood dental caries: a review," *BMC Oral Health,* vol. 19, no. 172, pp. 1-9, 2019.

[24] Braimoh.M, Ogunbodede.E, Adeniyi.A., "Integration of Oral Health into Primary Health Care System: Views of Primary Health Care Workers in Lagos State, Nigeria," *Journal of Public Health Africa,* vol. 5, no. 328, pp. 35-39, 2014.

[25] Adeniyi.A, Donnelly.L, Janssen.P, Jevitt.C, Kardeh.B, von Bergmann.H, Brondani.M., "Pregnant women's perspectives on integrating preventive oral health in prenatal care," *BMC Pregnancy and Childbirth,* vol. 21, no. 271, pp. 1-10, 2021.

[26] Petersen.PE, "Strengthening of Oral Health Systems: Oral Health through Primary Health Care," *Med Princ Pract. Global Oral Health Programme, Prevention of Non-Communicable Diseases, World Health Organization,,* vol. 23, no. suppl 1, pp. 3-9, 2014.

[27] Petersen.PE, Baez.RJ, Ogawa.H., "Global application of oral disease prevention and health promotion as measured 10 years after the 2007 World Health Assembly statement on oral health," *Community Dentistry and Oral Health Epidemiology,* vol. 48, no. 4, pp. 338-348, 2020.

[28] WHO, "World health statistics: a wealth of information on global public health," World Health Organization, 2013. [Online]. Available: http://www .who.int/gho/publications/world_health_statistics/2013/en/.. [Accessed 16th June 2018].

[29] Hess.RF, Gililland.CS, Dembélé.J., "Prevalence and Predictors of Periodontal Disease among Pregnant Women in Mali, West Africa," *Ann Med Health Sci Res,* vol. 7, pp. 263-270, 2017.

[30] Uwambaye.P, Kerr.M, Rulisa.S, Harlan.S, Munyanshongore.C., "Prevalence of Periodontitis and Associated Factors among Pregnant Women: A cross sectional survey in Southern Province, Rwanda," *Rwanda Journal of Medicine and Health Sciences ,* vol. 4, no. 1, pp. 131-150, 2021.

[31] Singh.S, Nazeer. J, Singh.R, Kavita.K, Iqubal.MA, Singh.R., “Predictors and prevalence of periodontitis among pregnant women of slum areas of Patna, India: An opportunity for oral health promotion,” *Journal of Education and Health Promotion,* vol. 10, no. 133, pp. 1-7, 2021.

[32] Mwangosi.IMEAT & Kiango.MM, “Oral health experience during pregnancy and dental service utilization in Bariadi District, Tanzania,” *Tanzania Journal of Health Research,* vol. 14, no. 2, pp. 1-7, 2012.

[33] FMoH, “National Oral Health Policy. National Oral Health Policy,” Calvary Digital Press Federal Ministry of Health., Abuja, Nigeria, 2012.

[34] Etiaba.E, Uguru.N, Ebenso.B, Russo.G, Ezumah.N, Uzochukwu.B, Onwujekwe.O., “Development of oral health policy in Nigeria: an analysis of the role of context, actors and policy process,” *BMC Oral Health,* vol. 15, no. 56, pp. 1-10, 2015.

[35] WHO, "Promoting Oral Health in Africa: Prevention and control of oral diseases and noma as part of essential non-communicable disease intervention.," World Health Organization, Africa Region, 2016.

[36] Braimoh.M, Ogunbodede.E, Adeniyi.A., “Integration of Oral Health into Primary Health Care System: Views of Primary Health Care Workers in Lagos State, Nigeria,” *J Public Health Africa,* vol. 5, no. 1, pp. 35-39, 2014.

[37] WHO, “World Health Organization(WHO). 2014. Non-Communicable Disease Risk Factor baseline Survey; Uganda 2014 Report,” World Health Organization, Kampala, Uganda, 2014.

[38] Wandera.M, Åstrøm.AN, Okullo.I, Tumwine.JK., “Determinants of periodontal health in pregnant women and association with infants’ anthropometric status: a prospective cohort study from Eastern Uganda,” *BMC Pregnancy and Childbirth,* vol. 12, no. 90, pp. 1-9, 2012.

[39] Muwazi.L,Rwenyonyi.CM,Nkamba.M,Mugyenyi.G,Kwizeru.G,Okullo.I., “Periodontal conditions, low birth weight and preterm birth among postpartum mothers in two tertiary health facilities in Uganda,” *BMC Oral Health.doi: 10.1186/1472-6831-14-42,* vol. 14, no. 42, pp. 1-8, 2014.

[40] MoH, “National Oral Health Policy,” World Health Organization, Kampala, Uganda, 2007.

[41] Creswell.JW, Research Design: Qualitative, Quantitative and Mixed Methods Approaches., Fourth Edition ed. Lincoln: . ed., California: SAGE Publications, Inc., 2014.

[42] George.A,Dahlen.HG, Reath.J, Ajwani.S, Bhole.S, Korda.A, Ng Chok.H, Miranda.C, Villarosa.A, Johnson.M., “What do antenatal care providers understand and do about oral health care during pregnancy: a cross-sectional survey in New South Wales, Australia,” *BMC Pregnancy and Childbirth,* vol. 16, no. 382, pp. 1-10, 2016.

[43] George, A.; Sousa, M.S.; Kong, A.C.; Blinkhorn, A.; Patterson Norrie, T.; Foster, J.; Dahlen, H.G.; Ajwani, S.; Johnson, M., “Effectiveness of preventive dental programs offered to mothers by non-dental professionals to control early childhood dental caries: a review,” *BMC Oral Health,* vol. 19, no. 172, pp. 1-9, 2019.

[44] Marzieh Araban.MD, Heydarabadi.AB, Cheraghian, B, Stein.LAR. , “Effectiveness of message-framing to improve oral health behaviors and dental plaque among pregnant women,” *Archives of Public Health,* vol. 79, no. 117, pp. 1-8, 2021.

[45] Heilbrunn-Lang.AY, de Silva.AM, Lang.G, George.A, Ridge.A, Johnson.M, Bhole.S, Gilmour.C., “Midwives’ perspectives of their ability to promote the oral health of pregnant women in Victoria, Australia,” *BMC Pregnancy and Childbirth,* vol. 15, no. 110, pp. 1-11, 2015.

[46] Converso.D, Sottimano.I, Guidetti.G, Loera.B, Cortini.M, Viotti.S., “Aging and Work Ability: The Moderating Role of Job and Personal Resources,” *Front. Psychol,* p. 8:2262, 2018.

[47] Al-Haroon.HI & Al-Qahtani.MF, “The demographic predictors of job satisfaction among the nurses of a major public hospital in KSA,” *J Taibah Univ Med Sci,* vol. 15, no. 1, p. 32–38, 2020.

[48] Ilmarinen. V, Ilmarinen. J, Huuhtanen. P, Louhevaara.V, Näsman. O. , “Examining the factorial structure, measurement invariance and convergent and discriminant validity of a novel self-report measure of work ability: work ability--personal radar,” *Economics,* vol. 58, no. 8, pp. 1445-60, 2015.

[49] George.A, Dahlen.HG, Reath.J,Ajwani.S, Bhole.S, Korda.A, Ng Chok.H, Miranda.C, Villarosa.A, Johnson.M., “What do antenatal care providers understand and do about oral health care during pregnancy: a cross-sectional survey in New South Wales, Australia,” *BMC Pregnancy and Childbirth.,* vol. 382 , no. 2016, pp. 1-10, 2016.

[50] Audeta.L, Bourgaulta.P, Rocheforta.CM., "Associations between nurse education and experience and the risk of mortality and adverse events in acute care hospitals: A systematic review of observational studies," *International Journal of Nursing Studies,* vol. 80, no. 2018, p. 128–146, 2018.

[51] Papastavrou.E, Efstathiou.G, Lemonidou.C, Kalafati.M, Katajisto.J, Suhonen.R., “Cypriot and Greek nurses' perceptions of the professional practice environment,” *Int Nurs Rev,* vol. 6, no. 2014, pp. 171-178, 2014.

[52] Stalpers. D, de Brouwer. BJ, Kaljouw.MJ, Schuurmans. MJ., “Association between characteristics of the nurse work environment and five nurse-sensitive patient outcomes in hospitals: a systematic review of literature,” *Int. J. Nurs. Stud,* vol. 52, no. 4, p. 817–835, 2015.

[53] Ayalew.F, Kibwana.S, Shawula.S, Misganaw.E, Abosse.Z, van Roosmalen.J, Stekelenburg.J,Mi Kim.Y, Teshome.M, Mariam.DW., “Understanding job satisfaction and motivation among nurses in public health facilities of Ethiopia: a cross-sectional study,” *BMC Nursing,* vol. 18, no. 46, pp. 1-13, 2019.

[54] Uwambaye.P, Munyanshongore.C, Kerr.M, Shiau.H, Nyiringango.G, Rulisa.S., “Assessment of the Knowledge, Attitude and Practices of Nurses and Midwives Working at Antenatal Clinics in the Southern Province of Rwanda on Periodontal Diseases: A Cross-Sectional Survey,” *Advances in Medical Education and Practice,* vol. 2020, no. 11, p. 517–523, 2020.

[55] Paneer.S, Muthusamy.N, Manickavel.RP, Venkatakrishnan.CJ, Rathnavelu.P, Jayaram.M., “Evaluation of Gynecologists' Awareness About Oral Health Condition During Pregnancy in Chennai City,” *J Pharm Bioallied Sci,* vol. 11, no. Suppl 2, p. S331–S334., 2019.

[56] Lee.JM & Shin.TJ, "Use of local anaesthetics for dental treatment during pregnancy; safety for parturient," *J Dent Anesth Pain Med,* vol. 17, no. 2, p. 81–90, 2017.

[57] Lee.J, Lee.J, Kim.HJ, Seo.K., "Dental anaesthesia for patients with allergic reactions to lidocaine: two case reports," *J Dent Anesth Pain Med,* vol. 16, no. 3, pp. 209-212, 2016.

[58] Favero.V, Bacci.C, Volpato.A, Bandiera. M, Favero. L, Zanette.G., "Pregnancy and Dentistry: A Literature Review on Risk Management during Dental Surgical Procedures," *Dent. J,* vol. 9, no. 46, pp. 1-16, 2021.

[59] Shah.D, Parsi.L, Bagher.S, Finkelman.M, Loo.C., "Effectiveness of an Oral Health Education Program for Obstetrician/Gynecologist Residents at Tufts Medical Center," *J Int Soc Prev Community Dent,* vol. 7, no. Suppl 2, p. S107–S112, 2017.

[60] Momanyi.GO, Adoyo.MA, Mwangi.EM, Mokua.DO., "Value of training on motivation among health workers in Narok County, Kenya," *Pan Afr Med J,* vol. 23, no. 261, pp. 1-12, 2016.

[61] Stuart.J, Hoang.H, Crocombe.L, Barnett.T., "Relationships between dental personnel and non-dental primary health care providers in rural and remote Queensland, Australia: dental perspectives," *BMC Oral Health,* vol. 17, no. 99, pp. 1-10, 2017.

[62] Johnson.M, Ajesh.G,Dahlen.H,Ajwani.S,Bhole.S,Blinkhorn.A., "The midwifery initiated oral health-dental service protocol: an intervention to improve oral health outcomes for pregnant women," *BMC Oral Health,* vol. 15, no. 2, pp. 1-9, 2015.

[63] Khanbodaghi.A, Natto.ZS, Forero.M, Loo.CY., "Effectiveness of interprofessional oral health program for pediatric nurse practitioner students at Northeastern University, United States," *BMC Oral Health,* vol. 19, no. 170, pp. 1-8, 2019.

[64] McDonald.J, Jayasuriya .R, Harris .MF., "The influence of power dynamics and trust on multidisciplinary collaboration: a qualitative case study of type 2 diabetes mellitus," *BMC Health Services Research,* vol. 12, no. 63, pp. 1-10, 2012.

[65] Harnagea.H, Lamothe.L,Couturier.H,Esfandiari.S,Voyer.R,Charbouneau.A,Emami.R., "From theoretical concepts to policies and applied prgrams:the landscaepe of integration of oral health in primary care," *BMC Oral Health,* vol. 18, no. 23, pp. 1-12, 2018.

[66] Douglass. AB, Douglass. JM, Krol. DM., "Educating Pediatricians and Family Physicians in Children's Oral Health.," *Academic Pediatrics. doi: 10.1016/j.acap,* vol. 9, no. 6, 2009.

[67] Schadewaldt.V, McInnes.E, Hiller.JE, Gardner.A., "Views and experiences of nurse practitioners and medical practitioners with collaborative practice in primary health care – an integrative review," *BMC Familty practice,* vol. 14, no. 132, pp. 1-11, 2013.

[68] Muthuri.RNDK, Senkubuge.F, Hongoro.C., "Determinants of Motivation among Healthcare Workers in the East African Community between 2009–2019: A Systematic Review," *Healthcare. doi:10.3390/healthcare8020164,* vol. 8, no. 164, pp. 1-24, 2020.

[69] Burke.W W & Litwin. G H, " A Causal Model of Organizational Performance and Change.. Journal of Management, 18(3), p.529.," *Journal of Management,* vol. 18, no. 3, pp. 523-545, 1992.

[70] Okello.DRO & Gilson .L, "Exploring the influence of trust relationships on motivation in the health sector: a systematic review," *Human Resources for Health,* vol. 13, no. 16, pp. 1-18, 2015.

[71] Figueroa.CA, Harrison.R, Chauhan.A, Meyer .L., "Priorities and challenges for health leadership and workforce management globally: a rapid review," *BMC Health Services Research,* vol. 19, no. 239, pp. 1-11, 2019.

[72] Shariff.N, "Factors that act as facilitators and barriers to nurse leaders' participation in health policy development," *BMC Nursing ,* vol. 13, no. 20, pp. 1-13, 2014.

[73] WHO, "Ottawa charter for health promotion. A commitment to Internatioanl Health Action," Ottawa, 1986.

[74] Johnson.M, George.A, Dahlen.H, Ajwani.S, Bhole.S, Blinkhorn.A, Ellis.S, Yeo.A., "The midwifery initiated oral health-dental service protocol: an intervention to improve oral health outcomes for pregnant women," *BMC Oral Health.http://www.biomedcentral.com/1472-6831/15/2,* vol. 15, no. 2, pp. 1-8, 2015.

Click here to access/download
Supporting Information
S1.sav

Factors influencing adoption of oral health promotion by antenatal care providers in Moyo district, North-Western Uganda

Patrick Madrama Lulu (MPH)[1], Miisa Nanyingi (MBChB, MSHSM)[2]

[1] Faculty of Health Sciences, Uganda Martyrs University, P.o. Box 5498, Kampala –Uganda.
Current address: Clarke International University, P.o. Box 7782, Kampala-Uganda.
[2]Faculty of Health Sciences, Uganda Martyrs University, P.o. Box 5498, Kampala -Uganda.

Corresponding Author: Patrick Madrama Lulu; Email:lulu.madrama@gmail.com; https://orcid.org/0000-0002-2838-8532

Key Words: *Adoption, Oral Health, Oral Health Promotion, Oral Hygiene, Antenatal care providers, Healthy Public Policy, perinatal, Periodontal Disease, UMU*

Abstract

Background: Oral health promotion(OHP) during pregnancy is an important global public health and basic human right issue related to quality of life. Several statements and guidelines have been published emphasizing the need for improved oral health care of pregnant mothers, prenatal care providers have missed this critical opportunity. In this study, we assessed factors influencing adoption of oral health promotion by Antenatal care providers.

Materials and Methods: A descriptive cross-sectional study design that employed both quantitative and qualitative data collection methods and analysis. 152 samples determined using Yamane's 1967 and stratified sampling technique was used. Three FGDs and six KI interviews were held. Univariate, bivariate and multivariate analyses were done using SPSS (20.0) and ATLAS Ti for qualitative analysis.
Results: Adoption of OHP was low 28% (42). Factors influencing adoption were age of respondents (aOR=0.066, 95%CI=0.009-0.465, p= 0.006*), (p=0.032*), level of care of health facility (aOR=0.050, 95%CI=0.008-0.322, p= 0.002*), (p=0.004*), good understanding between dentists and ANC providers (aOR=0.283, 95%CI=0.084-0.958, p= 0.042*), availability of practice guideline for OHP in ANC (aOR=0.323, 95%CI=0.108-0.958, p= 0.043*), number of years at work (p=0.084), being knowledgeable (aOR=2.143, 95%CI=0.864-5.311, p= 0.100), having skills to advance OHP(aOR=0.734, 95%CI=0.272-1.984, p= 0.542), Management being good at influencing new practices (aOR=00.477.734, 95%CI=0.227-2.000, p= 0.477). More emphasis on national and local of oral health issues, continuous staff training on oral health, dissemination of National oral health policy (NOHP) were some of key issues that emerged from the qualitative results.

Conclusion: Adoption of OHP was low. This was attributed to age, number of years spent at work, level of health facility, having good understanding between dentists and ANC providers, availability of practice guidelines, dissemination of National oral health policy, continuous staff training. We recommend the current NOHP to be reviewed, develop prenatal OHC guidelines, enhance the capacity of ANC providers through training, collaboration with dentists and launch official adoption of OHP.

1. Background

Oral disease is one of the most important public health issues in the world with significant socio-economic impacts [1, 2, 3, 4]. Among pregnant women, the common oral health diseases are periodontal diseases with a high prevalence rate [5]. Oral health is a basic human right fundamental to people's quality of life [7], achieving oral health goals would require health systems being supported by enabling actions in education, improved nutrition and hygiene [8]. Antenatal care is the care provided by skilled healthcare professionals to pregnant women and adolescent girls to ensure the best health conditions for both mother and baby during pregnancy and its components include; risk identification, prevention and management of pregnancy-related or concurrent diseases including health education and health promotion [9]. Pregnancy has an impact on oral related quality of life [10]. Pregnancy provides an opportunity for women to initiate new healthy behaviours, including oral health practices, dental and prenatal care providers have missed this critical opportunity to promote oral health [6]. Some of the missed opportunities for oral health promotion include; oral health education, oral health examination and referral for dental services when mothers are receiving antenatal care.

Oral health is a basic human right fundamental to people's quality of life [7], achieving oral health goals would require health systems being supported by enabling actions in education, improved nutrition and hygiene [8]. Antenatal care is the care provided by skilled healthcare professionals to pregnant women and adolescent girls to ensure the best health conditions for both mother and baby during pregnancy and its components include; risk identification, prevention and management of pregnancy-related or concurrent diseases including health education and health promotion [9]. Pregnancy has an impact on oral related quality of life [10].

Globally, several statements and guidelines have been published emphasizing the need for improved oral health care of pregnant mothers, because, hormonal changes during pregnancy combined with neglected oral hygiene tend to increase the incidence of oral diseases including gingivitis [11]. The United States

Formatted: Font: Not Bold, Font color: Black

of America, Department of Health and Human Services highlighted the need for partnerships between dentists and other health professionals, including nurses and midwives and these recommendations have resulted in the development of evidence-based practice guidelines for oral health care during pregnancy and early childhood for all health professionals including prenatal care providers (American Academy of Pediatric Dentistry, 2009; California Dental Association Foundation, American College of Obstetricians and Gynecologists, 2010; National Maternal and Child Oral Health Resource Center, 2008; [12].

In low and middle-income countries, the prevalence of oral diseases continues to increase, moreover, tooth decay is rapidly increasing among adults and there will be a huge burden of this health problem in the future if sustainable programs are not put in place [13, 14]. The good oral health of a pregnant woman should be considered to be of utmost importance for dental practitioners and antenatal care providers [15, 16, 17, 18]. Integration of Oral health into primary care has been implemented in some health care systems to reduce the burden of oral health disease and to improve access to oral health care, especially for disadvantaged populations and communities [19, 20, 21]. Asia developed a strategy in which emphasis was put on the integration of oral health promotion and other disease prevention strategies [22], various countries have implemented preventive strategies to maintain the oral health of mothers during pregnancy [23].

Adopting an integrated approach to healthcare would achieve better outcomes for patients with oral diseases and several policy recommendations have been made including the adoption of oral health in all policies, strengthening inter-professional collaboration, and inclusion of oral health in the curriculum for all healthcare professionals [24, 25]. In addition, the WHO global policy for the improvement of oral health showed that the promotion of oral health is a cost-effective strategy to reducing the burden of oral diseases and maintaining oral health and quality of life. Oral Health services should focus on prevention and early diagnosis [26, 27].

In Sub-Saharan Africa, there is an increased prevalence of dental diseases, resulting from increased consumption of sugars and inadequate exposure to fluorides [28], with an increased burden of periodontal diseases among pregnant women in Mali [29, 30, 31], while pregnant mothers in Tanzania had increased burden of bleeding gum, dental pain, tooth decay and swollen gums [32]. Nigeria's National oral health policy indicated the need for oral health to be included as a component of health promotion and must be provided in antenatal care services [33, 34] and emphasis has been placed on integrating oral health promotion in maternal and child care among African Countries [35, 36].

In Uganda, the prevalence of oral diseases among women was the highest at 42.4% compared to the general population [37], 67.0% of pregnant mothers having periodontal diseases [38], 86.0% postpartum mothers with plague deposits [39]. Uganda has emphasised that appropriate promotion of oral health requires integration of oral health policy elements, strategies and policies of all sectors to impact community health, including maternal and child health [40]. In the Moyo district, there is limited data on the level and factors associated with the adoption of Oral health promotion by antenatal care providers.

2. Materials and Methods

2.1 Study Population and settings

This cross sectional study on adoption of oral health promotion was done among antenatal care providers working in general hospital, Health Centre IVs and health centre IIIs within Moyo district between august 2017 to October 2017.

The inclusion criteria required that, for the ANC providers to participate in the study, they should be health workers working at ANC units, those who had ever worked at the ANC unit, including all Nurses, Midwives, Clinical Officers and medical doctors within the selected health facilities who consented to participate in the study. We excluded all ~~health professionals who were not~~ Nurses, Midwives, Clinical Officers, Gynaecologists, paediatricians, general medical doctors and had never worked at the ANC department before the study and all ANC providers who had spent less than six months at work at the time this study was done.

2.2 Study variables

Adoption of Oral Health Promotion as a dependent variable was measured using 24 items of practices related to oral health care services by antenatal care providers, with a total score of mean of 18 (75%, SD 3.2) and a greater proportion of ANC providers had not adopted oral health promotion during ANC.~~.~~ ~~ANC providers who mentioned doing up to three quarters 3/4 (75%) or more than 75% of the 24 practice items were considered to have adopted oral health promotion for pregnant mothers. Using scale of 0-17 (0-70%) items to refer to non-adoption and scale 18-24 (75%-100%) referred to adoption of oral health promotion by ANC providers.~~

Knowledge and attitude influencing adoption of oral health promotion: 11 items were used to measure the level of knowledge, with a ~~of the respondents on adoption of oral health promotion~~mean score of correct answers 7 (64%, SD 2.2)~~. ANC providers who scored less or equal to 6(≤55%) were recorded as not knowledgeable, those who scored 7 and above (≥64%) as knowledgeable.~~ For measuring attitude of the respondents, 6 items were used, with a mean ~~those who~~ score~~d~~ 4~~3~~ (66~~≤50~~%, SD 0.98). ~~and below were recorded to have had negative attitude and respondents who scored 4 to 6 (≥50%) were recorded to have positive attitude on adoption of oral health promotion.~~

Capacity of Health facilities to provide oral health promotion activities: 16 items were used to measure the capacity through binary scale (no or yes) ~~of the health facilities to promote oral health during antenatal care~~ and 11 items used to measure the **strategies required to influencing adoption of oral health promotion**, through binary scale (no or yes) ~~by antenatal care providers~~there were inadequate strategies to influencing oral health promotion by ANC providers.~~.~~

2.3 Study Design

A descriptive cross-sectional study was used where both quantitative and qualitative methods of data collection and analysis were employed. A concurrent data collection approach was used including information and the interpretation of overall results [41], this design was used because of the shorter data

collection period and the need to provide a comprehensive analysis of the research problem. 152 samples were determined using Yamane's (1967) formula $n = \frac{N}{1+N(e)^2}$, Where n = sample size, N = Number of Nurses, Midwives, Clinical Officers and Medical Doctors in the selected health facilities within Moyo district (245), e = Level of precision or sampling error (0.05).and a stratified sampling technique was used. Three FGDs and six KI interviews were held. Univariate, bivariate and multivariate analyses were done using SPSS (20.0) for quantitative analysis and ATLAS Ti for qualitative analysis.

2.2 Data Analysis

We analysed Quantitative data using Microsoft Excel 2010 and SPSS 20. Chi-square statistics were computed to check for statistically significant differences in the parameters between the dependent and independent variables (Univariate, bivariate and multivariate analysis were performed in SPSS version 20.0). Logistic regression was done to obtain strength of association between categorical dependent and independent variables and statistically significant items at this level were then analysed using multivariate logistic regression to obtain adjusted conclusions for the finding of the study. Qualitative data were transcribed verbatim, coded and organised in themes in ATLAS.ti. Triangulation was done to incorporate both the quantitative and qualitative data obtained from respondents.

Commented [MP1]: Statistically significant values at this level have been clearly shown in table 2 of this current manuscript.

3. Results

3.1 Demographic Characteristics of the respondents

Our study considered a total of 152 ANC providers, mean age 36, range 37 years, a standard deviation of 10.49, of which, 57(37.5%) were 37 years and above, 92(60.5%) females, 106(69.9%) had worked between 1-10 years, 82(53.9%) working HC IIIs, 67(44.1%) Nurses and 82(53.9%) were Certificate holders. ~~42 (28%) of the respondents out of 152 had adopted oral health promotion for pregnant mothers.~~

Although individual practices of ANC providers appeared to be below the expected level of good practice in the adoption of oral health promotion, the management and officer in charge of the MCH programme noted that adoption or integration of oral health promotion in ANC would be vital for overcoming some of the challenges of poor oral health, its outcome on pregnancy and the child (See ***Table 1***).

> *"There is need to adopt or integrate oral health promotion into ANC services to overcome some of the challenges created by the poor oral health of the pregnant mothers" (KI 1).*

More emphasis supporting the need for adoption of Oral Health promotion by ANC providers showed that adoption is likely to reduce heart diseases among pregnant mothers;

> *"Of late there are increased cases of heart diseases…… originating from the dental issues as bacteria tend to move to the bloodstreams and later on move to the heart valves without knowing, adopting oral health promotion will have a possibility of reducing such challenges in the future" KI 2).*

> *"It will be cheaper adopting oral health promotion into ANC services compared to when it handled as a separate program" (FGD 1).*

Table 1: Demographic Characteristics of respondents

**Variables**	**Number (%)**<br>**N=(Total =152(%))**	**Pe[illegible]age**<br>**(T[illegible] 100%)**
**Age**		
22-26<br>27-31<br>32-36<br>37 & above	29(19.1)<br>33(21.7)<br>33(21.7)<br>57(37.5)	19.1<br>21.7<br>21.7<br>37.5
**Gender**		
Male<br>Female	60(39.5)<br>92(60.5)	39.5<br>60.5
**Number of years at work**		
1-10 years	106(69.7)	69.79
11-20 years	23(15.1)	15.1
21-30 years	8(5.3)	5.3
31-40 years	15(9.9)	9.9
**Level of Health Facility**		
Hospital	40(26.3)	26.3
HCIV	30(19.7)	19.7
HCIII	82(54)	543.9
**Professional Cadre**		

Nurses	67(44.1)	44.1
Midwives	35(23.0)	23.0
Double trained Nurse/midwives	24(15.8)	15.8
Clinical Officers	2013.2)	13.2
Medical doctors	6(3.9)	3.9
**Professional Qualification**		
Certificate	82(53.9)	53.9
Diploma	60 (39.5)	39.5
Bachelor’s Degree	10(6.6)	6.6

3.2 Level of adoption of Oral Health Promotion by ANC Providers

42 (28%) of the respondents out of 152 had adopted oral health promotion for pregnant mothers. Although individual practices of ANC providers appeared to be low,below the expected level of good practice in the adoption of oral health promotion, the management and officer in charge of the MCH programme noted that adoption or integration of oral health promotion in ANC would be vital for overcoming some of the challenges of poor oral health, its outcome on pregnancy and the child (See figure 1).

> *"There is need to adopt or integrate oral health promotion into ANC services to overcome some of the challenges created by the poor oral health of the pregnant mothers" (KI 1).*

More emphasis supporting the need for adoption of Oral Health promotion by ANC providers showed that adoption is likely to reduce heart diseases among pregnant mothers;

> *“Of late there are increased cases of heart diseases…… originating from the dental issues as bacteria tend to move to the bloodstreams and later on move to the heart valves without knowing, adopting oral health promotion will have a possibility of reducing such challenges in the future” KI 2).*

> *"It will be cheaper adopting oral health promotion into ANC services compared to when it handled as a separate program" (FGD 1).*

Figure 1: Level of adoption of Oral Health Promotion by ANC Providers

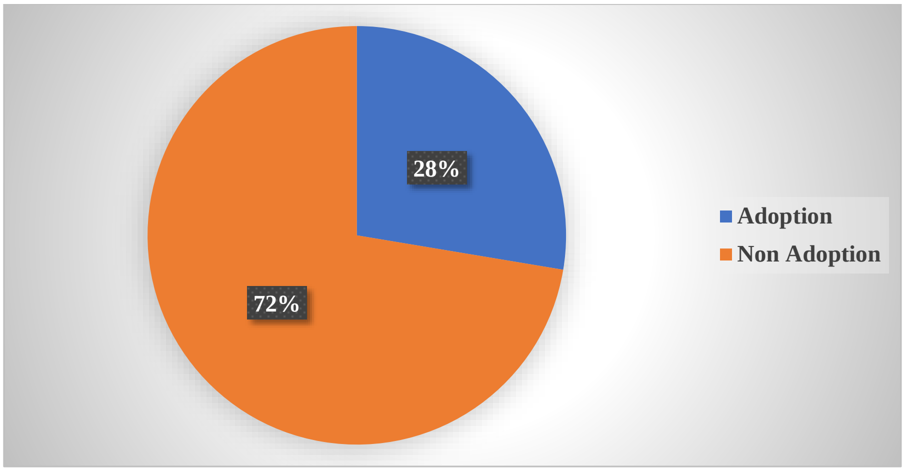

3.3~~2~~ Factors influencing adoption of Oral Health Promotion by ANC Providers

In our multivariate analysis, age of respondents in years 22-26 (aOR=0.066, 95%CI=0.009-0.465, p= 0.006*~~ ~~)~~(p=0.032*)~~, level of care of health facility (aOR=0.050, 95%CI=0.008-0.322, p= 0.002*) ~~(p=0.004*)~~, ~~ ~~good understanding between dentists and ANC providers (aOR=0.283, 95%CI=0.084-0.958, p= 0.042*), availability of practice guideline for OHP in ANC (aOR=0.323, 95%CI=0.108-0.958, p= 0.043*), worked 1-10 years (p=0.011*), were statistically significant and associated with adoption of oral health promotion by ANC providers. However, general number of years at work (p=0.084), being knowledgeable (aOR=2.143, 95%CI=0.864-5.311, p= 0.100), having skills to advance OHP in ANC (aOR=0.734, 95%CI=0.272-1.984, p= 0.542), Management good at influencing new practices (aOR=00.477.734, 95%CI=0.227-2.000, p= 0.477) were not statistically significantly associated with adoption oral health promotion by ANC providers (see ***Table 2***).

> *"Top management at the national level is not good at influencing new policies and guidelines, there is no dissemination of the National Oral health policy, if dental health issues are not marketed by the authorities, no one will pay attention and it will continue to be neglected, and yet adoption will lead to a reduction on*

missed opportunities, maximizing resources – one staff offering all that is needed and improving service delivery" (KI 1).

In our univariate analysis, female ANC providers were more likely to adopt oral health promotion compared to their male counterparts. Respondents aged between 22-26 years~~, 27- 31 years, and 32-36 years~~ were less likely to adopt oral health promotion during prenatal care compared to those aged 37 years and above. Respondents who had been working for the last 21-30 years were 14 times more likely to adopt oral health promotion compared to response who had for 31-40 years~~, 6 times more likely compared to those for 27- 31 years, and 8 times more likely to those who had for 22-26 years respectively~~ (see ***Table 2***).

In terms of the level of health facilities, respondents working at the ~~district general hospital~~health centre IV were less likely to adopt oral health promotion compared to those working at HC IIIs. ~~However, working at a general hospital contributed to 82.7% adoption compared to working at HCIV which only contributed to 13.8% adoption of oral health promotion by ANC providers.~~

Knowledgeable respondents were four times more likely to adopt oral health promotion compared to those that were not knowledgeable and respondents who had no skills to advance OHP in ANC were less likely to adopt oral health promotion compared to ~~those~~ respondents who ~~said they~~ had no skills in advancing OHP for prenatal care.

Respondents who said that management was good at influencing new practices, were more likely to adopt oral health promotion compared to those who said that management was not good at influencing new practices. Having no g~~G~~ood understanding between dentists and ANC providers less likely contributed to the adoption of OHP, and having no practice guidelines for OHP by ANC providers less likely~~would~~ le~~a~~d to the adoption of oral health promotion~~. However, dental health issues appear to be neglected by the authorities compared to other departments~~ (See ***Table 2***).

"Even if the staff members make recommendations for some dental items, the oral health department issues are always pushed aside and not taken care of" (KI 3).

The whole district only offers extraction within government health facilities, other services are in a private dental clinic (KI 4).

We do not have oral health policies for ANC and there is no local guideline that is put in place yet. We do not have the National Oral Health policy even in our dental department and I have never seen it before" (KI 2).

There is a need for training of ANC providers on oral health promotion for pregnant mothers (FGD 1).

The ANC providers were also asked to raise issues that should be included in the oral health promotion guideline. More than half 95 (62.5%) of the respondents mentioned almost all responses and these were; prevention of oral diseases and this was followed by oral health education 93 (61.2%), Oral health education 93 (61.6%), ways of improving access to oral healthcare 91(60.3%) and assessment of pregnant women's oral health 88 (58.3%) (See ~~***Figure 1***~~Figure 2).

Figure 2: Multiple responses showing issues to be included in oral health promotion guideline for ANC

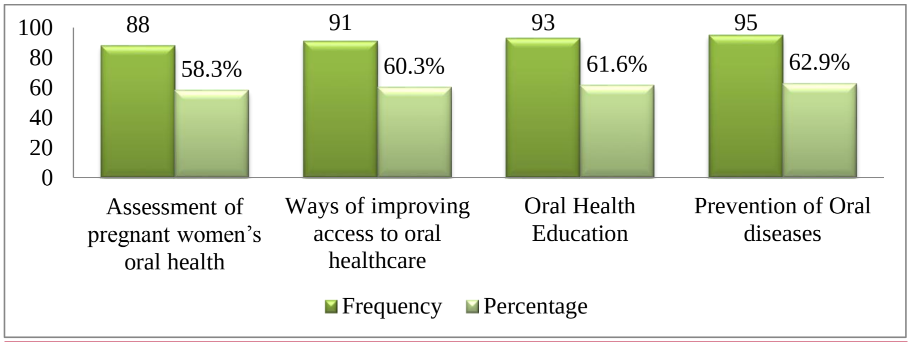

In line with participants' knowledge on the kind of preventive oral care pregnant women should receive the majority 124 (89.9%) of the respondents mentioned how to maintain oral hygiene, 57(41.3%) of the ANC providers mentioned education on nutrition, less than half 45(32.6%) and 29(21.0%) of the ANC providers mentioned oral health assessment as well as referral, only 2(1.4%) mentioned other preventive care services as the kind of preventive services pregnant women should receive (See Figure 3).

Formatted: Font: Not Bold

Figure ~~1~~3:Knowledge on the kind of preventive oral care pregnant women should receive

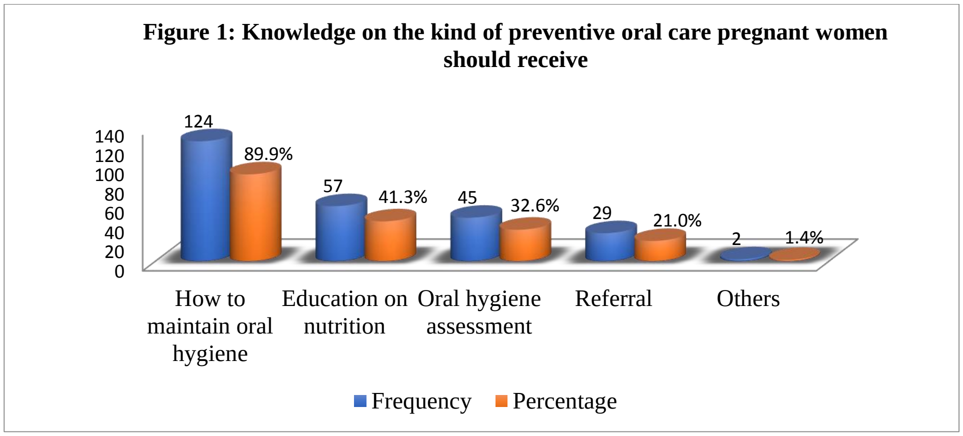

In line with the knowledge of respondents on the safety of obtaining dental radiographs during pregnancy, the majority 125 (82.2%) of the respondents were not aware that it is safe to obtain dental radiographs in pregnant women while only 26 (17.1%) of the respondents said obtaining dental radiograph in pregnant women is safe (See ***Figure*** 4).

Figure ~~2~~4:Awareness on the safety of obtaining dental radiographs during pregnancy

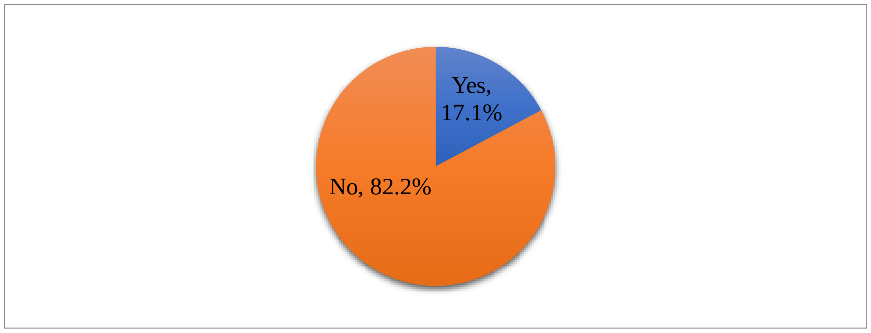

~~The ANC providers were also asked to raise issues that should be included in the oral health promotion guideline. More than half 95 (62.5%) of the respondents mentioned almost all responses and these were;~~

~~prevention of oral diseases and this was followed by oral health education 93 (61.2%), Oral health education 93 (61.6%), ways of improving access to oral healthcare 91(60.3%) and assessment of pregnant women's oral health 88 (58.3%) (See ***Figure 3***).~~

~~Figure 3: Multiple responses showing issues to be included in oral health promotion guideline for ANC~~

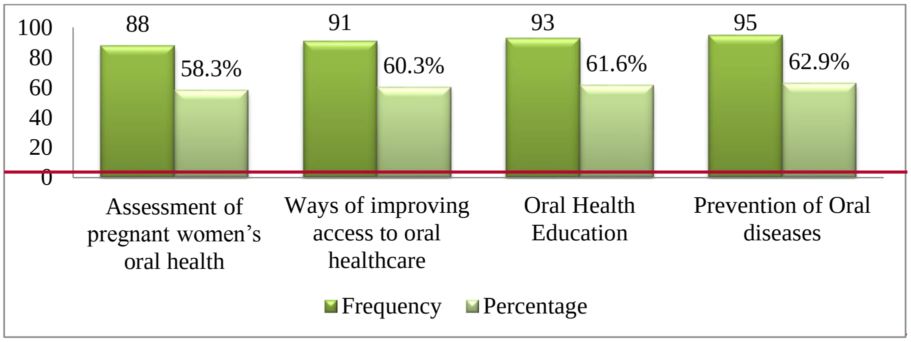

Table 2:Factors influencing adoption of oral health promotion by ANC providers in Moyo district, north-western Uganda

	**Adoption of Oral Health Promotion by ANC Providers**		**Univariate Logistic Regression Analysis**		**Multivariate Logistic Regression Analysis**	
**Variables**	**Adoption (%)**	**Non-Adoption (%)**	**UOR (95%CI)**	**P-value**	**AOR (95%CI)**	**P-value**
**Age in years**					~~**8.779**~~	~~**0.032***~~
22-26	3	26	0.184(0.050-0.679)	0.011*	0.066(0.009-0.465)	**0.006***
27-31	8	25	0.509(0.195-1.327)	0.167	0.148(0.029-0.741)	**0.020***
32-36	2	24	0.597(0.235-1.517)	0.278	0.130(0.026-0.642)	0.084
37 & above	22	35	1		1	
**Number of years at work**					~~**6.648**~~	~~**0.084**~~
1-10 years	1	14	5.273(0.663-41.920)	0.116	25.241(2.075-307.041)	**0.011***
11-20 years	29	17	7.467(0.825-67.573)	0.074	8.205(0.749-89.852)	0.085
21-30 years	8	15	14.000(1.200-163.367)	0.035*	12.064(0.775-187.885)	0.075
31-40 years	4	4	1		1	
**Level of Care**					~~**10.969**~~	~~**0.004***~~
Hospital	2	28	0.827(0.366-1.869)	0.647	0.317(0.097-1.034)	0.057*
HC IV	12	28	0.138(0.031-0.621)	0.010*	0.050(0.008-0.322)	**0.002***
HCIII	28	54	1		1	
**Knowledge**						
Knowledgeable	7	49	4.016(1.642-9.823)	0.002*	2.143(0.864-5.311)	0.1000

Not Knowledgeable	35	61	1		1	
**Have skills to advance OHP in ANC**						
No	12	54	0.415(0.193-0.893)	0.024*	0.734(0.272-1.984)	0.542
Yes	30	56	1		1	
**Management is good at influencing new practices**						
No	7	44	0.300(0.122-0.735)	0.008*	0.477(0.227-2.000	0.477
Yes	35	66	1		1	
**A good understanding exists between dentists and ANC providers**						
No	6	47	0.223(0.087-0.674)	0.002*	0.283(0.084-0.958)	**0.042***
Yes	36	63	1		1	
**Practice guidelines for OHP in ANC are available**						
No	28	54	0.482(0.229-1.013)	0.054*	0.323(0.108-0.958)	**0.043***
Yes	14	56	1		1	

Discussion

Adoption of Oral Health Promotion by Respondents

In this study, adoption of oral health promotion by ANC providers was low 42 (28%) of 152. Similarly, a study conducted in Australia among antenatal care providers showed that very few ANC providers were involved in practices that promote oral health among pregnant women [42]. Adoption of oral health by ANC providers would play a significant role not only in the lives of pregnant mothers and also in their babies [43]. Maternal oral health behaviour could be shaped during antenatal care [44], promotion of oral health among pregnant women by Nurses would be feasible and need to be incorporated into the first antenatal booking visit and also recognized that oral health promotion was within their scope of practice [45].

Respondents aged between 22-26 years, 27- 31 years, and 32-36 years were less likely to adopt oral health promotion during prenatal care compared to those aged 37 years and above. There was a statistically significant relationship between age and adoption of oral health promotion. This may be due

to experiences gained over the years about oral health. Contrary to the findings of our study, another study noted that age is significantly and negatively associated with workability [46], job satisfaction among Nurses is also related to age [47].

Moreover, the number of years one spent at work improves occupational competence, consists of expertise, skills, and knowledge acquired through experience, training, and education, people's work and are influenced by previous work experiences [48]. Our study noted that respondents who had been working for the last 21-30 years were 14 times more likely to adopt oral health promotion compared to response who had for 31-40 years, who were 6 times more likely compared to those for 27- 31 years, and 8 times more likely to those who had for 22-26 years respectively. Similarly, [49] noted in their study that, there was an association between years spent at work and adoption of oral health promotion, Nurses 'experiences are closely related to the occurrence of adverse events [50]. However, Positive work environments are important in achieving patient safety, quality care and favourable patient outcomes [51].

Similarly, experience give Nurses' exposure to different patient or health-related conditions, including different clinical and preventive scenarios that would eventually contribute to increased knowledge, critical thinking and technical skills [52]. In general, job performance among nurses is motivated by years of experience [53]. However, it has also been noted that age and length of time in service within ANC do not influence knowledge of oral healthcare for prenatal mothers [54].

In terms of the level of the health facility where respondents were working, our study found that those working at the district general hospital were less likely to adopt oral health promotion compared to respondents working at HC IIIs. However, working at a general hospital contributed to 82.7% adoption compared to working at HCIV which only contributed to 13.8% adoption of oral health promotion by ANC providers. This may be because most of the care of HCIIIs in the district are offered by generally Nurses, Midwives and Clinical officers, ANC at this level of facility handles all aspects of preventive

services including oral health education and promotion. The general hospital and HCIVs may be overwhelmed by several conditions because of referrals from lover facilities.

In our study, less than half 56(36.8%) of the respondents were knowledgeable about OHP in ANC and those knowledgeable were four times more likely to adopt oral health promotion compared to those that were not knowledgeable. Similarly, Nurses and Midwives had insufficient knowledge of oral healthcare [54]. Respondents in our study were not knowledgeable about the fact that several physiological changes take place among pregnant women and these have an effect on oral health, pregnancy and brings about several changes in the oral cavity [55]. Our finding was similar to the study in Australia which noted that ANC providers had limited knowledge on prenatal oral healthcare [49].

In terms of knowledge on the safety of dental procedures during pregnancy, our study found that ANC providers were not knowledgeable about the safety of different dental services that pregnant women should receive, including the use and safety of local anaesthetics during pregnancy, teeth extraction, scaling and Root planning and Root Canal. This knowledge about the safety of dental procedures during pregnancy should be addressed to improve service utilization and oral health promotion among pregnant mothers. Similarly, a study by [55] found a knowledge gap among gynaecologists about the safety of some of the dental procedures for pregnant mothers. Local anesthetics lidocaine commonly used during dental care are safe, although, with some negligible adverse effect [56], some of these effects may include immune response-mediated allergic reactions and others that are unrelated to the immune response and these allergic responses are very rare in practice [57].

About the safety of obtaining dental radiographs in pregnant women, the majority 125 (82.2%) of the ANC providers were not aware that it is safe to obtain dental radiographs in pregnant women and use of dental radiographs, if necessary, after the first trimester does not pose any risk to the developing fetus [58], however, it is recommended to use protective lead aprons and thyroid collar to shield the sensitive areas. This kind of misconception could however be overcome among ANC providers by continuous

services professional training and collaboration among different professionals within the different health facilities.

In our study, respondents who had no skills to advance OHP in ANC were less likely to adopt oral health promotion compared to those who said they had skills in advancing OHP for prenatal care. This calls for the need to incorporate prenatal oral care in health higher institutions of learning and continuous professional education for ANC providers in practice. Health institutions have limited training for prenatal oral healthcare [59], adequate skills attained by health workers would enable them to perform desired tasks, more regular training and paying attention to discuss career development prospects would be of great value to advance their skills [60].

In addition, our study found that having a good understanding between dentists and ANC providers contributed to adoption. A study conducted in Australia found no interprofessional collaboration and understanding between non-dentists and dentists [61]. An inter-professional collaboration education model for dental and medical providers are needed and the provision of an appropriate referral system for comprehensive clinical care of pregnant patients and accredited standards that encourage development and implementation [63, 64]. There is a need for collaboration between ANC providers with dentists to encourage all pregnant women to comply with the oral health professionals' recommendations regarding appropriate dental brushing techniques and the importance of dental visits [63]. However, Collaboration across organizational boundaries remains challenging due to power dynamics and trust that affect the strategic choices made by each health professional about whether to collaborate, with whom and to what level [64]. These decisions directly affect inter-professional relationships trust and respect can be fostered through a mix of interventions aimed at building personal relationships and establishing agreed rules that govern collaborative care and that are perceived as fair to all professions. Some of the factors that facilitate the integration of oral health are interdisciplinary education and collaborative practices between dental and other healthcare professionals [65]. Adoption

of oral promotion requires coordination and cooperation between ANC providers and dentists [66]. Understanding and interprofessional collaboration improve professional output, through the provision of clarity around the meaning and definition of professional roles [67].

In terms of management, respondents who said management was not good at influencing new practices among ANC providers were less likely to adopt oral health promotion compared to management being good at influencing new practices. Poor leadership were a key factor leading to providers' weak workplace trust and contributed to often-poor quality services, driving a perverse cycle of negative patient-provider relations [71]. For leaders to have lasting influence, staff members should be involved in health policy development and some of the key participatory roles in health policy development include mentoring, supporting and developing future policymakers [72].

In addition, the Ottawa charter emphasized that all sectors at all levels be involved in formulating policy and direct them to be aware of the health consequences of their decisions and to accept their responsibilities for health, health promotion policy requires the identification of obstacles to the adoption of healthy public policies and ways of removing them, thus health policies must focus on making it easy for beneficiaries to make healthier choices [73]. Involvement of staff members would help them on what is in the policy and also improve on policy dissemination and implementation at all levels, avoiding situations wherein a district, some oral health professionals have not even seen the national oral health policy until it was presented to them during the research work.

Moreover, our study found that there is a need to develop oral health promotion guidelines for pregnant mothers and according to [49], many countries have developed oral health guidelines for pregnant mothers to help them improve the oral health of both pregnant mothers and the babies and the main barriers for ANC providers in promoting oral health was lack of practice guidelines on oral health care during pregnancy. There is a need for local and national health policy agenda in Uganda to incorporate the prevention of oral diseases during pregnancy [38]. Burke-Litwin model noted that, when staff

members feel that organizational policies and procedures are favourable to them, they are motived to perform the required actions, implying that appropriate formulation and implementation of policies motivate staff members to perform required actions.

Study Strength and Limitations

Our study had several strengths firstly, the large sample size covering three quarters of the health workers and all categories of cadres of health workers in the district, secondly, our study was the first in Uganda with a focus on the adoption of oral health promotion by antenatal care providers, we involved research assistants who were well trained to create rapport with respondents, thirdly, we trained research assistants on data collection procedures, maintaining confidentiality, ensure correctness and consistency of recorded data. Fourth, all our research tools were fully pretested and checked for accuracy and correctness, only filled questionnaires were included for analysis. However, our study could not be used to analyse behaviour or practices of the ANC providers over some time as it was a snapshot, limitation of confounding bias which was controlled by restricting participation to only those who had worked for at least six months and above and participation to only ANC provider who had ever worked or are working at ANC department in order ~~running stepwise multiple logistic regression analysis~~ to arrive at meaningful and conclusive results.

Conclusion

Adoption of OHP was low. Factors influencing the adoption of oral health promotion were, Age of respondents, the number of years at work, good understanding between dentists and ANC providers, and management being good at influencing new practices contributed to an increased level in the adoption of oral health promotion. However, knowledge gap, poor dissemination of National oral health policy and lack of prenatal oral health guidelines were some of the barriers to an increased level of adoption by antenatal care providers. We, therefore, recommend that the current NOHP should be reviewed, develop

prenatal OHC guidelines, enhance the capacity of ANC providers through training, collaboration with dentists and launch official adoption of OHP.

Ethical Considerations

This study was conducted in accordance with the Helsinki declaration guideline, a waiver was granted by International Health Sciences University Research Ethics Committee (IHSU-REC) (IHSU-REC/004). Prior to the waiver, administrative clearance was done Uganda Martyrs University Scientific Review Committee, a written consent was obtained from Moyo District Health Office (DHO), verbal consent from the medical superintendent of Moyo hospital, in-charges of the Dental unit, Antenatal care department and various health facilities under study and finally from participants under study.

Abbreviations

ANC: Antenatal care; aOR: Adjusted Odds Ratio; CI: Confidence Interval; MoH: Ministry of Health; FDG: Focus Group Discussion; KI: Key Informants; uOR: Unadjusted Odds Ratio; UMU: Uganda Martyrs University; HSSIP: Health Sector Strategic and Investment Plan; WHO: World Health Organization.

Acknowledgement

We are grateful to Uganda Martyrs University for awarding the primary author with a master degree in Public Health – Health Promotion. We thank International Health Sciences University (Currently, Clarke International University) research ethics committee for according us a waiver for our protocol. we appreciate all our research assistants for the support rendered during the process of data collection.

Authors Contribution

PML and MN conceptualised and designed the study. PML obtained the needed data, PML analysed the data and interpreted the results, PLM drafted and made the final manuscript, PLM and MN performed critical revisions of the manuscript. Both PML and MN approved the final manuscript.

Funding

There was no funding by any organization, this research was carried out under the supervision of Uganda Martyrs University Faculty of Health Sciences as a requirement for the award of masters of Public Health- Health Promotion for the first author.

Availability of data and materials

The used data set or data set used during the analysis of this current study are available from the corresponding author on reasonable request.

Consent for publication

No application

Competing interests

The authors declare that they do not have any competing interests.

References

[1] Marcenes.W, Kassebaum.NJ, Bernabe.E, Flaxman.A, Neghavi.M, Lopez.A, Murray.CJL., "Global Burden of oral conditions in 1990-2010: A systematic Analysis.," *.J Dent Res.DOI:10.1177/0022034513490168,* vol. 92, no. 7, pp. 592-595, 2013.

[2] Kassebaum.N, Hernandez.C, Bailey.J, Hay.SI, James.S,Mokdad.A, Nixon.M., "Global, Regional, and National Levels and Trends in Burden of Oral Conditions from 1990 to 2017: A Systematic Analysis for the Global Burden of Disease 2017 Study," *J Dent Res* ~~*Journal of Dental Research*~~, 2020.

[3] WHO, "Oral health. Key facts," World Health Organization, Geneva, 2020.

[4] Kassebaum, N.J., Smith, A.G., Bernabé, E., Fleming, T.D., Reynolds, A.E., Vos, T., Murray, C.J.L., Marcenes, W. and GBD 2015 Oral Health Collaborators., "Global, regional, and national prevalence, incidence, and disability-adjusted life-years for oral conditions for 195 countries, 1990–2015: a systematic analysis for the global burden of diseases, injuries, and risk factors," *Journal of dental research,* vol. 96, no. 4, pp. 380-387, 2017.

Formatted: Font: Italic

[5] Salih.Y, Nasr.AM, Ahmed.ABA, Sharif.ME, Adam.I., “Prevalence of and risk factors for periodontal disease among pregnant women in an antenatal care clinic in Khartoum, Sudan,” *BMC Research Notes,* vol. 13, no. 147, pp. 1-5, 2020.

[6] Marchi.KS, Rinki.C, Shah.M, Dove.M, Terpak .C, Curtis.MP, Braveman.P., “Medical Provider Promotion of Oral Health and Women's Receipt of Dental Care During Pregnancy,” *Jmch~~ournal of Maternal and Child Health~~.doi: 10.1007/s10995-018-02714-z. ,* vol. 23, no. 7, pp. 890-902, 2019.

[7] Martínez.GR & Albuquerque.A, “The right to oral health in the Liverpool Declaration,” *Rev. bioét. (Impr.),* vol. 25, no. 2, pp. 224-33, 2017.

[8] Muhoozi.GK, Atukunda.P, Skaare.AB, Willumsen.T, Diep.LM, Westerberg.AC, Iversen.PO., “Effects of nutrition and hygiene education on oral health and growth among toddlers in rural Uganda: follow-up of a cluster-randomised controlled trial,” *Trop Med Int Health,* vol. 23, no. 4, pp. 391-404, 2018.

[9] WHO, "WHO recommendations on antenatal care for a positive pregnancy experience.," World Health Organization, 2016.

[10] Fakheran.O, Keyvanara.M, Saied-Moallemi .Z, Khademi.A., “The impact of pregnancy on women’s oral health-related quality of life: a qualitative investigation,” *BMC Oral Health,* vol. 20, no. 294, pp. 1-11, 2020.

[11] Harnagea.H,Lamothe.L,Couturier.H,Esfandiari.S,Voyer.R,Charbouneau.A,Emami.R., “From theoretical concepts to policies and applied prgrams:the landscaepe of integration of oral health in primary care,” *BMC Oral Health,* vol. 18, no. 23, pp. 1-12, 2018.

[12] Northridge. ME, Kumar.A, Kaur.R., “Disparities in Access to Oral Health Care,” *Annual Review of Public Health,* p. 513–35, 3 January 2020.

[13] WHO, "Prevention is better than treatment," Bull World Health Organ, 2015.

[14] WHO, “Oral health. Key facts,” World Health Organization, Geneva, 2020.

[15] Ramamurthy.J & Irfana.F, "Assessment of knowledge and awareness about periodontal oral health among pregnant women-A Questionnaire study," *Int J Cur Res Rev,* vol. 9, no. 1, pp. 9-12, 2017.

[16] Yenen.Z & Ataçağ.T,, “Oral care in pregnancy,” *J Turk Ger Gynecol Assoc 2019,* vol. 20, no. 4, pp. 264-8, 2019.

[17] Fakheran.O, Keyvanara.M, Saied-Moallemi.Z, Khademi.A., “The impact of pregnancy on women’s oral health-related quality of life: a qualitative investigation,” *BMC Oral Health.https://doi.org/10.1186/s12903-020-01290-5,* vol. 20, no. 294, pp. 1-11, 2020.

[18] García-Pola.M, González-Díaz.A, García-Martín.JM., “Effect of a Preventive Oral Health Program Starting during Pregnancy: A Case-Control Study Comparing Immigrant and NativeWomen and Their Children,” *J.Environ. Res. Public Health,* Vols. 18,, no. 4096, pp. 1-14, 2021.

[19] Petersen.PE, Baez.RJ, Ogawa.H., “Global application of oral disease prevention and health promotion as measured 10 years after the 2007 World Health Assembly statement on oral health,” *Community Dental and Oral Health Epidemiology,* vol. 48, no. 4, pp. 338-348, 2020.

[20] Emami.E, Harnagea.H, Girard.F, Charbonneau.A, Voyer.R, Bedos.CP, Chartier.M, Wootton.J, Couturier.Y., “Integration of oral health into primary care: a scoping review protocol,” *BMJ Open doi:10.1136/bmjopen-2016-013807,* vol. 10, no. 1136, pp. 1-5, 2016.

[21] Braimoh.M, Ogunbodede.E, Adeniyi.A., “Integration of oral health into primary health care system: views of primary health care workers in Lagos State, Nigeria,” *Journal of Public Health in Africa,* vol. 5, no. 328, pp. 35-39, 2014.

[22] WHO, "Formulating Oral Health strategy for South-East Asia. Report of a Regional Consultation," World Health Organization, South Asia; India, Chiang Mai, Thailand, 2009.

[23] Ajesh.G, Sousa.MS, Kong.AC, Blinkhorn.A, Norrie.TP, Foster.J, Dahlen. HG, Ajwani.S, Johnson.M., “Effectiveness of preventive dental programs offered to mothers by non-dental professionals to control early childhood dental caries: a review,” *BMC Oral Health,* vol. 19, no. 172, pp. 1-9, 2019.

[24] Braimoh.M, Ogunbodede.E, Adeniyi.A., “Integration of Oral Health into Primary Health Care System: Views of Primary Health Care Workers in Lagos State, Nigeria,” *Journal of Public Health Africa,* vol. 5, no. 328, pp. 35-39, 2014.

[25] Adeniyi.A, Donnelly.L, Janssen.P, Jevitt.C, Kardeh.B, von Bergmann.H, Brondani.M., “Pregnant women’s perspectives on integrating preventive oral health in prenatal care,” *BMC Pregnancy and Childbirth,* vol. 21, no. 271, pp. 1-10, 2021.

[26] Petersen.PE, “Strengthening of Oral Health Systems: Oral Health through Primary Health Care,” *Med Princ Pract. Global Oral Health Programme, Prevention of Non-Communicable Diseases, World Health Organization,,* vol. 23, no. suppl 1, pp. 3-9, 2014.

[27] Petersen.PE, Baez.RJ, Ogawa.H., “Global application of oral disease prevention and health promotion as measured 10 years after the 2007 World Health Assembly statement on oral health,” *Community Dentistry and Oral Health Epidemiology,* vol. 48, no. 4, pp. 338-348, 2020.

[28] WHO, “World health statistics: a wealth of information on global public health,” World Health Organization, 2013. [Online]. Available: http://www .who.int/gho/publications/world_health_statistics/2013/en/.. [Accessed 16th June 2018].

[29] Hess.RF, Gililland.CS, Dembélé.J., “Prevalence and Predictors of Periodontal Disease among Pregnant Women in Mali, West Africa,” *Ann Med Health Sci Res,* vol. 7, pp. 263-270, 2017.

[30] Uwambaye.P, Kerr.M, Rulisa.S, Harlan.S, Munyanshongore.C., “Prevalence of Periodontitis and Associated Factors among Pregnant Women: A cross sectional survey in Southern Province, Rwanda,” *Rwanda Journal of Medicine and Health Sciences ,* vol. 4, no. 1, pp. 131-150, 2021.

[31] Singh.S, Nazeer. J, Singh.R, Kavita.K, Iqubal.MA, Singh.R., “Predictors and prevalence of periodontitis among pregnant women of slum areas of Patna, India: An opportunity for oral health promotion,” *Journal of Education and Health Promotion,* vol. 10, no. 133, pp. 1-7, 2021.

[32] Mwangosi.IMEAT & Kiango.MM, “Oral health experience during pregnancy and dental service utilization in Bariadi District, Tanzania,” *Tanzania Journal of Health Research,* vol. 14, no. 2, pp. 1-7, 2012.

[33] FMoH, “National Oral Health Policy. National Oral Health Policy,” Calvary Digital Press Federal Ministry of Health., Abuja, Nigeria, 2012.

[34] Etiaba.E, Uguru.N, Ebenso.B, Russo.G, Ezumah.N, Uzochukwu.B, Onwujekwe.O., “Development of oral health policy in Nigeria: an analysis of the role of context, actors and policy process,” *BMC Oral Health,* vol. 15, no. 56, pp. 1-10, 2015.

[35] WHO, "Promoting Oral Health in Africa: Prevention and control of oral diseases and noma as part of essential non-communicable disease intervention.," World Health Organization, Africa Region, 2016.

[36] Braimoh.M, Ogunbodede.E, Adeniyi.A., “Integration of Oral Health into Primary Health Care System: Views of Primary Health Care Workers in Lagos State, Nigeria,” *J Public Health Africa,* vol. 5, no. 1, pp. 35-39, 2014.

[37] WHO, “World Health Organization(WHO). 2014. Non-Communicable Disease Risk Factor baseline Survey; Uganda 2014 Report,” World Health Organization, Kampala, Uganda, 2014.

[38] Wandera.M, Åstrøm.AN, Okullo.I, Tumwine.JK., “Determinants of periodontal health in pregnant women and association with infants’ anthropometric status: a prospective cohort study from Eastern Uganda,” *BMC Pregnancy and Childbirth,* vol. 12, no. 90, pp. 1-9, 2012.

[39] Muwazi.L,Rwenyonyi.CM,Nkamba.M,Mugyenyi.G,Kwizeru.G,Okullo.I., “Periodontal conditions, low birth weight and preterm birth among postpartum mothers in two tertiary health facilities in Uganda,” *BMC Oral Health.doi: 10.1186/1472-6831-14-42,* vol. 14, no. 42, pp. 1-8, 2014.

[40] MoH, “National Oral Health Policy,” World Health Organization, Kampala, Uganda, 2007.

[41] Creswell.JW, Research Design: Qualitative, Quantitative and Mixed Methods Approaches., Fourth Edition ed. Lincoln: . ed., California: SAGE Publications, Inc., 2014.

[42] George.A,Dahlen.HG, Reath.J, Ajwani.S, Bhole.S, Korda.A, Ng Chok.H, Miranda.C, Villarosa.A, Johnson.M., “What do antenatal care providers understand and do about oral health care during pregnancy: a cross-sectional survey in New South Wales, Australia,” *BMC Pregnancy and Childbirth,* vol. 16, no. 382, pp. 1-10, 2016.

[43] George, A.; Sousa, M.S.; Kong, A.C.; Blinkhorn, A.; Patterson Norrie, T.; Foster, J.; Dahlen, H.G.; Ajwani, S.; Johnson, M., “Effectiveness of preventive dental programs offered to mothers by non-dental professionals to control early childhood dental caries: a review,” *BMC Oral Health,* vol. 19, no. 172, pp. 1-9, 2019.

[44] Marzieh Araban.MD, Heydarabadi.AB, Cheraghian, B, Stein.LAR. , "Effectiveness of message-framing to improve oral health behaviors and dental plaque among pregnant women," *Archives of Public Health,* vol. 79, no. 117, pp. 1-8, 2021.

[45] Heilbrunn-Lang.AY, de Silva.AM, Lang.G, George.A, Ridge.A, Johnson.M, Bhole.S, Gilmour.C., "Midwives' perspectives of their ability to promote the oral health of pregnant women in Victoria, Australia," *BMC Pregnancy and Childbirth,* vol. 15, no. 110, pp. 1-11, 2015.

[46] Converso.D, Sottimano.I, Guidetti.G, Loera.B, Cortini.M, Viotti.S., "Aging and Work Ability: The Moderating Role of Job and Personal Resources," *Front. Psychol,* p. 8:2262, 2018.

[47] Al-Haroon.HI & Al-Qahtani.MF, "The demographic predictors of job satisfaction among the nurses of a major public hospital in KSA," *J Taibah Univ Med Sci,* vol. 15, no. 1, p. 32–38, 2020.

[48] Ilmarinen. V, Ilmarinen. J, Huuhtanen. P, Louhevaara.V, Näsman. O. , "Examining the factorial structure, measurement invariance and convergent and discriminant validity of a novel self-report measure of work ability: work ability--personal radar," *Economics,* vol. 58, no. 8, pp. 1445-60, 2015.

[49] George.A, Dahlen.HG, Reath.J,Ajwani.S, Bhole.S, Korda.A, Ng Chok.H, Miranda.C, Villarosa.A, Johnson.M., "What do antenatal care providers understand and do about oral health care during pregnancy: a cross-sectional survey in New South Wales, Australia," *BMC Pregnancy and Childbirth.,* vol. 382 , no. 2016, pp. 1-10, 2016.

[50] Audeta.L, Bourgaulta.P, Rocheforta.CM., "Associations between nurse education and experience and the risk of mortality and adverse events in acute care hospitals: A systematic review of observational studies," *International Journal of Nursing Studies,* vol. 80, no. 2018, p. 128–146, 2018.

[51] Papastavrou.E, Efstathiou.G, Lemonidou.C, Kalafati.M, Katajisto.J, Suhonen.R., "Cypriot and Greek nurses' perceptions of the professional practice environment," *Int Nurs Rev,* vol. 6, no. 2014, pp. 171-178, 2014.

[52] Stalpers. D, de Brouwer. BJ, Kaljouw.MJ, Schuurmans. MJ., "Association between characteristics of the nurse work environment and five nurse-sensitive patient outcomes in hospitals: a systematic review of literature," *Int. J. Nurs. Stud,* vol. 52, no. 4, p. 817–835, 2015.

[53] Ayalew.F, Kibwana.S, Shawula.S, Misganaw.E, Abosse.Z, van Roosmalen.J, Stekelenburg.J,Mi Kim.Y, Teshome.M, Mariam.DW., "Understanding job satisfaction and motivation among nurses in public health facilities of Ethiopia: a cross-sectional study," *BMC Nursing,* vol. 18, no. 46, pp. 1-13, 2019.

[54] Uwambaye.P, Munyanshongore.C, Kerr.M, Shiau.H, Nyiringango.G, Rulisa.S., "Assessment of the Knowledge, Attitude and Practices of Nurses and Midwives Working at Antenatal Clinics in the Southern Province of Rwanda on Periodontal Diseases: A Cross-Sectional Survey," *Advances in Medical Education and Practice,* vol. 2020, no. 11, p. 517–523, 2020.

[55] Paneer.S, Muthusamy.N, Manickavel.RP, Venkatakrishnan.CJ, Rathnavelu.P, Jayaram.M., "Evaluation of Gynecologists' Awareness About Oral Health Condition During Pregnancy in Chennai City," *J Pharm Bioallied Sci,* vol. 11, no. Suppl 2, p. S331–S334., 2019.

[56] Lee.JM & Shin.TJ, "Use of local anaesthetics for dental treatment during pregnancy; safety for parturient," *J Dent Anesth Pain Med,* vol. 17, no. 2, p. 81–90, 2017.

[57] Lee.J, Lee.J, Kim.HJ, Seo.K., "Dental anaesthesia for patients with allergic reactions to lidocaine: two case reports," *J Dent Anesth Pain Med,* vol. 16, no. 3, pp. 209-212, 2016.

[58] Favero.V, Bacci.C, Volpato.A, Bandiera. M, Favero. L, Zanette.G., "Pregnancy and Dentistry: A Literature Review on Risk Management during Dental Surgical Procedures," *Dent. J,* vol. 9, no. 46, pp. 1-16, 2021.

[59] Shah.D, Parsi.L, Bagher.S, Finkelman.M, Loo.C., "Effectiveness of an Oral Health Education Program for Obstetrician/Gynecologist Residents at Tufts Medical Center," *J Int Soc Prev Community Dent,* vol. 7, no. Suppl 2, p. S107–S112, 2017.

[60] Momanyi.GO, Adoyo.MA, Mwangi.EM, Mokua.DO., "Value of training on motivation among health workers in Narok County, Kenya," *Pan Afr Med J,* vol. 23, no. 261, pp. 1-12, 2016.

[61] Stuart.J, Hoang.H, Crocombe.L, Barnett.T., "Relationships between dental personnel and non-dental primary health care providers in rural and remote Queensland, Australia: dental perspectives," *BMC Oral Health,* vol. 17, no. 99, pp. 1-10, 2017.

[62] Johnson.M, Ajesh.G,Dahlen.H,Ajwani.S,Bhole.S,Blinkhorn.A., "The midwifery initiated oral health-dental service protocol: an intervention to improve oral health outcomes for pregnant women," *BMC Oral Health,* vol. 15, no. 2, pp. 1-9, 2015.

[63] Khanbodaghi.A, Natto.ZS, Forero.M, Loo.CY., "Effectiveness of interprofessional oral health program for pediatric nurse practitioner students at Northeastern University, United States," *BMC Oral Health,* vol. 19, no. 170, pp. 1-8, 2019.

[64] McDonald.J, Jayasuriya .R, Harris .MF., "The influence of power dynamics and trust on multidisciplinary collaboration: a qualitative case study of type 2 diabetes mellitus," *BMC Health Services Research,* vol. 12, no. 63, pp. 1-10, 2012.

[65] Harnagea.H, Lamothe.L,Couturier.H,Esfandiari.S,Voyer.R,Charbouneau.A,Emami.R., "From theoretical concepts to policies and applied prgrams:the landscaepe of integration of oral health in primary care," *BMC Oral Health,* vol. 18, no. 23, pp. 1-12, 2018.

[66] Douglass. AB, Douglass. JM, Krol. DM., "Educating Pediatricians and Family Physicians in Children's Oral Health.," *Academic Pediatrics. doi: 10.1016/j.acap,* vol. 9, no. 6, 2009.

[67] Schadewaldt.V, McInnes.E, Hiller.JE, Gardner.A., "Views and experiences of nurse practitioners and medical practitioners with collaborative practice in primary health care – an integrative review," *BMC Familty practice,* vol. 14, no. 132, pp. 1-11, 2013.

[68] Muthuri.RNDK, Senkubuge.F, Hongoro.C., "Determinants of Motivation among Healthcare Workers in the East African Community between 2009–2019: A Systematic Review," *Healthcare. doi:10.3390/healthcare8020164,* vol. 8, no. 164, pp. 1-24, 2020.

[69] Burke.W W & Litwin. G H, " A Causal Model of Organizational Performance and Change.. Journal of Management, 18(3), p.529.," *Journal of Management,* vol. 18, no. 3, pp. 523-545, 1992.

[70] Okello.DRO & Gilson .L, "Exploring the influence of trust relationships on motivation in the health sector: a systematic review," *Human Resources for Health,* vol. 13, no. 16, pp. 1-18, 2015.

[71] Figueroa.CA, Harrison.R, Chauhan.A, Meyer .L., "Priorities and challenges for health leadership and workforce management globally: a rapid review," *BMC Health Services Research,* vol. 19, no. 239, pp. 1-11, 2019.

[72] Shariff.N, "Factors that act as facilitators and barriers to nurse leaders' participation in health policy development," *BMC Nursing ,* vol. 13, no. 20, pp. 1-13, 2014.

[73] WHO, "Ottawa charter for health promotion. A commitment to Internatioanl Health Action," Ottawa, 1986.

[74] Johnson.M, George.A, Dahlen.H, Ajwani.S, Bhole.S, Blinkhorn.A, Ellis.S, Yeo.A., "The midwifery initiated oral health-dental service protocol: an intervention to improve oral health outcomes for pregnant women," *BMC Oral Health.http://www.biomedcentral.com/1472-6831/15/2,* vol. 15, no. 2, pp. 1-8, 2015.

Patrick Madrama Lulu,
Clarke International University,
P.0. Box 7782, Kampala- Uganda
14th .11.2022

Dear Emily Chenette,
Editor in Chief, Plos One Journal

Ref: Response to Reviewers for "Factors influencing adoption of oral health promotion by antenatal care providers in Moyo district, North-Western Uganda."

Thank you for taking time to review our manuscript that was submitted to you, thank you for the comments. Below are the responses to the reviewers.

Section	Reviewers' Comment	Responses
**Abstract**	you may need to insert the OR and CIF for the first 2 factors as well	OR and CIF have been inserted for the first 2 factors.
	Delete a	a, have been deleted from aOR
**Background**	This sentence better to come after the second sentence in the introduction as it is continuation regarding oral health followed by the sentence starting with Antenatal care	The suggested sentence has been inserted after the second sentence in the introduction as suggested by the reviewers.
**Study Population and settings**	Exclusion criteria is to be revised! as a general principle exclusion is to exclude the already included (fulfilling the inclusion criteria) but with some characteristics they are to be excluded.	Exclusion criteria has been revised following general principles.
**Study Variables**	What was the logic behind applying these cut-off points? I would suggest to use the mean score as the cut-off point!	The researchers have now clearly indicated the values for mean scores for each cut off point.
	what was the cut-off point in these two variables	The cut-off point for these two variables have now been clearly indicated.
**Study Design**	Could you please elaborate on the equation by adding the numbers used in the equation e.g. population size	The formula for calculating the sample size has been elaborated as suggested by the reviewers and population size indicated.
**Data Analysis**	what was the level of significance used?	This has been adjusted.

**Results**	This is not shown in the table 1	Has been adjusted as required.
	what is the expected level and the actual level measured?	It was an error, expected levels have been removed.
	This table would be better presented in two columns the second containing both frequency and parentage!	This table 1 has been adjusted as suggested by reviewers.
	This should be 69.7<br>the total adds to 100.2%	This value has been adjusted in table 1, as required.
	This should be 54%	This has as well been adjusted as required.
	You need to mention the OR and CI	OR and CI have been added.
	ONLY this age-group was significant others were not<br>check the OR and CI AND p-values as well!	This has been adjusted as required.
	this comparison is wrong!!!<br>the reference group is 31-40 years' experience both groups are compared to the last group of experience (they are not significant look at te CI) REVISE	This has been revised.
	This comparison is wrong the reference group is HC IV	This has been adjusted.
	Wrong interpretation of the UOR!!<br>REVISE	The interpretation has been revised.
	compared to those had skills!!	This comparison has been adjusted.
	Mention the livelihood between the two groups UOR three times, eight times and twice	The likelihood between the two groups have been revised.
	This sentence belong to the discussion than the results section	The sentence has been removed from results section.
	These results depicted in figure 3 NOT one	This has been adjusted as required.
	These results are depicted in figure 1.	Has been adjusted as required.
	What was the CI	This has been revised in table 3.
**Strength and Limitations**	Data analyses does not control for any bias!	This has been revised.
**References**	Any abbreviation for this journal!	Abbreviation for the journals have been indicated as required.

Yours Sincerely

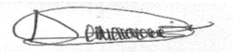

Patrick Madrama Lulu
Phone: +256 782 98 04 51
Email:Lulu.madrama@gmail.com
Consultant Research Ethics Committee/Lecturer, Clarke International University
