# Supplementary material for: Factors influencing adoption of oral health promotion by antenatal care providers in Moyo district, North-Western Uganda
Source: PLoS One. 2023 Apr 25;18(4):e0272238. doi: 10.1371/journal.pone.0272238 (PMC10128944; doi:10.1371/journal.pone.0272238)
Supplement: Attachment — Submitted filename: Response to Reviewers.pdf [file pone.0272238.s005.pdf]

Patrick Madrama Lulu,
Clarke International University,
P.0. Box 7782, Kampala- Uganda
25th .02.2023

Dear Emily Chenette,
Editor in Chief, Plos One Journal

Ref: Response to Reviewers for "Factors influencing adoption of oral health promotion by antenatal care providers in Moyo district, North-Western Uganda."

Thank you for taking time to review our manuscript that was submitted to you, thank you for the comments. Below are the responses to the reviewers.

Section	Reviewers' Comment	Responses
**Materials and Methods**	Please elaborate about your exclusion criteria	We were looking at the fact that Medical Doctors, Nurses, Clinical Officers etc who did not work at ANC department did not have much opportunity to care for pregnant mothers, therefore, did not qualify for the study.<br>ANC providers who had worked within the district less than six months may not be able to give authentic responses related to the study within the area of the study.
**Study Variables**	Items used to measure adoption: list of items used???	Items have been listed.
	please mention the list of practice items that was used	Items have been listed.
	List of items used to measure Knowledge	Items have been listed.
	List of items used to measure attitude	Items have been listed.
	List of items used to measure capacity of health facilities.	Items have been listed.
**Results**	please reformat this table, title for the column is missing in Table 1, has unlabelled column	This has been formated and deleted in line comments from previous first review.
	Clinical Officers - 2013.2 in table 1	This has been corrected 20(13.2)
	what was your basis for this conclusion??<br>How was the adoption measured?	Adoption was measured as described under the variables in page 4 of this manuscript.

General	Please address the comments from one of the reviewers who has asked to review English language and back your analysis with more graph(s) where necessary.	Review of English language have been done.<br>Our manuscript has the necessary supporting figures and tables in line with plos one guidelines.
	Methods section please revise the informed consent statement to reflect whether written/verbal informed consent was obtained from all participants for inclusion in the study.	This has been revised.

Yours Sincerely

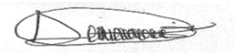

Patrick Madrama Lulu
Phone: +256 782 98 04 51
Email:Lulu.madrama@gmail.com
Consultant Research Ethics Committee/Lecturer, Clarke International University
