# Supplementary material for: Factors influencing adoption of oral health promotion by antenatal care providers in Moyo district, North-Western Uganda
Source: PLoS One. 2023 Apr 25;18(4):e0272238. doi: 10.1371/journal.pone.0272238 (PMC10128944; doi:10.1371/journal.pone.0272238)
Supplement: Attachment — Submitted filename: Response to Reviewers.docx [file pone.0272238.s003.docx]

Patrick Madrama Lulu,

Clarke International University,

P.0. Box 7782, Kampala- Uganda

14^th^ .11.2022

Dear Emily Chenette,

Editor in Chief, Plos One Journal

**Ref:** **Response to Reviewers for “Factors influencing adoption of oral health promotion by antenatal care providers in Moyo district, North-Western Uganda.”**

Thank you for taking time to review our manuscript that was submitted to you, thank you for the comments. Below are the responses to the reviewers.

| **Section** | **Reviewers’ Comment** | **Responses** |
| --- | --- | --- |
| **Abstract** | you may need to insert the OR and CIF for the first 2 factors as well | OR and CIF have been inserted for the first 2 factors. |
|  | Delete a | a, have been deleted from aOR |
| **Background** | This sentence better to come after the second sentence in the introduction as it is continuation regarding oral health followed by the sentence starting with Antenatal care | The suggested sentence has been inserted after the second sentence in the introduction as suggested by the reviewers. |
| **Study Population and settings** | Exclusion criteria is to be revised!  as a general principle exclusion is to exclude the already included (fulfilling the inclusion criteria) but with some characteristics they are to be excluded. | Exclusion criteria has been revised following general principles. |
| **Study Variables** | What was the logic behind applying these cut-off points?  I would suggest to use the mean score as the cut-off point! | The researchers have now clearly indicated the values for mean scores for each cut off point. |
|  | what was the cut-off point in these two variables | The cut-off point for these two variables have now been clearly indicated. |
| **Study Design** | Could you please elaborate on the equation by adding the numbers used in the equation e.g. population size | The formula for calculating the sample size has been elaborated as suggested by the reviewers and population size indicated. |
| **Data Analysis** | what was the level of significance used? | This has been adjusted. |
| **Results** | This is not shown in the table 1 | Has been adjusted as required. |
|  | what is the expected level and the actual level measured? | It was an error, expected levels have been removed. |
|  | This table would be better presented in two columns the second containing both frequency and parentage! | This table 1 has been adjusted as suggested by reviewers. |
|  | This should be 69.7  the total adds to 100.2% | This value has been adjusted in table 1, as required. |
|  | This should be 54% | This has as well been adjusted as required. |
|  | You need to mention the OR and CI | OR and CI have been added. |
|  | ONLY this age-group was significant others were not  check the OR and CI AND p-values as well! | This has been adjusted as required. |
|  | this comparison is wrong!!!  the reference group is 31-40 years’ experience both groups are compared to the last group of experience (they are not significant look at te CI) REVISE | This has been revised. |
|  | This comparison is wrong the reference group is HC IV | This has been adjusted. |
|  | Wrong interpretation of the UOR!!  REVISE | The interpretation has been revised. |
|  | compared to those had skills!! | This comparison has been adjusted. |
|  | Mention the livelihood between the two groups UOR three times, eight times and twice | The likelihood between the two groups have been revised. |
|  | This sentence belong to the discussion than the results section | The sentence has been removed from results section. |
|  | These results depicted in figure 3 NOT one | This has been adjusted as required. |
|  | These results are depicted in figure 1. | Has been adjusted as required. |
|  | What was the CI | This has been revised in table 3. |
| **Strength and Limitations** | Data analyses does not control for any bias! | This has been revised. |
| **References** | Any abbreviation for this journal! | Abbreviation for the journals have been indicated as required. |

Yours Sincerely

Patrick Madrama Lulu

Phone: +256 782 98 04 51

Email:Lulu.madrama@gmail.com

Consultant Research Ethics Committee/Lecturer, Clarke International University
