# Supplementary material for: Factors influencing adoption of oral health promotion by antenatal care providers in Moyo district, North-Western Uganda
Source: PLoS One. 2023 Apr 25;18(4):e0272238. doi: 10.1371/journal.pone.0272238 (PMC10128944; doi:10.1371/journal.pone.0272238)
Supplement: Attachment — Submitted filename: Reviewer Comments 24 Aug .docx [file pone.0272238.s001.docx]

**Reviewer Comments:**

Dear Authors,

I appreciated the entirety of your research article, it comprehends several aspects that are frequently assessed for different diseases in the oral cavity during pregnancy and antenatal care. Overall details shared in the article are adequately explained via appropriate tables ad figures. The present manuscript is well written, organized and the topic is interesting for the leadership of oral diseases. Minor changes are recommended to improve the manuscript of this article.

**Comment-1**

**Abstract:**

Write key words in capital letters or small letters. (Page-1)

**Comment-2**

**Background:**

Rephrase the lines 8 – 10 to make them more clear**.** Oral health is ---------- nutrition and Hygiene.

**Comment-3**

**Background:**

Mention the hormonal changes in line-16 (Page-2)

**Comment-4**

**Background:**

Correct the sentence: in all healthcare professionals around the globe. This study can be added in line-16 (Page-3) with the references 24,25.

1. Study, K. A. P., & Access, O. (2022). Emergency Management Readiness of Pakistani Dentists at Public Sector Teaching Hospitals. *Pakistan Journal of Medicine and Dentistry*, *11*(1), 78–84. https://doi.org/10.36283/pjmd11-1/013

**Comment-5**

**2.3 Study Design: Page-5**

How 152 samples using Yamane's (1967) were determined? Give an explanation?
